# Influence of voltine ecotype and geographic distance on genetic and haplotype variation in the Asian corn borer

**DOI:** 10.1002/ece3.7829

**Published:** 2021-07-09

**Authors:** Yangzhou Wang, Kyung Seok Kim, Qiyun Li, Yunyue Zhang, Zhen‐Ying Wang, Brad Steven Coates

**Affiliations:** ^1^ Northeast Agricultural Research Center of China Jilin Academy of Agricultural Sciences Changchun China; ^2^ Natural Resource Ecology and Management Iowa State University Ames IA USA; ^3^ Institute of Plant Protection Chinese Academy of Agricultural Sciences Beijing China; ^4^ Corn Insects and Crop Genetics Research Unit USDA ARS Ames IA USA

**Keywords:** divergent phenotypic selection, ecological adaptation, gene flow, population genetics

## Abstract

Diapause is an adaptive dormancy strategy by which arthropods endure extended periods of adverse climatic conditions. Seasonal variation in larval diapause initiation and duration in *Ostrinia furnacalis* may influence adult mating generation number (voltinism) across different local environments. The degree to which voltine ecotype, geographic distance, or other ecological factors influence *O. furnacalis* population genetic structure remains uncertain. Genetic differentiation was estimated between voltine ecotypes collected from 8 locations. Mitochondrial haplotypes were significantly different between historically allopatric univoltine and bivoltine locations, but confounded by a strong correlation with geographic distance. In contrast, single nucleotide polymorphism (SNP) genotypes show low but significant levels of variation and a lack of influence of geographic distance between allopatric voltine locations. Regardless, 11 of 257 SNP loci were predicted to be under selection, suggesting population genetic homogenization except at loci proximal to factors putatively under selection. These findings provide evidence of haplotype divergent voltine ecotypes that may be maintained in allopatric and sympatric areas despite relatively high rates of nuclear gene flow, yet influence of voltinism on maintenance of observed haplotype divergence remains unresolved.

## Graphical abstract

The Asian corn borer (ACB) is a major pest insect of cultivated maize throughout China, including Jilin Province where ecotypes of this species differ in voltine traits. The distribution of significant mitochondrial haplotype and nuclear genetic variation among sample locations suggests geography and voltinism are factors influencing contemporary ACB genetic structure in Jilin Province.

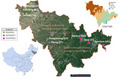

## 1 INTRODUCTION

The scope of adaptive mechanisms by which species respond to variation in their local environment presents fundamental questions for ecological and evolutionary studies. Cumulative effects of exposures to light (photoperiod) and heat (degree day accumulation) impact species growth rates and modify seasonal responses (Andrewartha & Birch, 1954). Thus, phenological and/or life cycle adaptations theoretically move ecotype variants toward trait accumulations that optimize survival and reproduction under a set of local environmental conditions (Fielding et al., 1999; Viegas et al., 2012). Among insects, these adaptations are critical for seasonal synchrony with host availabilities, capacity to complete development, or entrance into a state of dormancy prior to the onset of adverse seasonal conditions (Tauber & Tauber, 1986, 1981). Adaptive mechanisms modify diapause response to variable local environmental conditions including photoperiod and thermal gradients (Hut et al., 2013) and are manifested in differences in the number of generations per year (voltinism;Huang et al., 2013; Showers, 1981). Specifically, shifts from insect populations predominantly with a fixed single generation per year (univoltine) to those showing plasticity in environmentally determined generation number (multivoltine) generally follow latitudinal gradients. Regardless, disentangling the concurrent contributions of geographic and ecotype variation on allopatric population differentiation remains difficult.

The genus *Ostrinia* (Lepidoptera: Crambidae) is comprised of at least 20 species, many of which have diverse ecologically adapted and phenotypically diverged subspecies or ecotypes (Mutuura & Munroe, 1970; Ohno, 2003). Species with a preponderance of intraspecific phenotypic variants reside within the phylogenetic clade, Group III, which includes the highly related Asian corn borer, *O. furnacalis*, European corn borer, *O. nubilalis*, and adzuki bean borer, *O. scapulalis* (Kim et al., 1999). These divergent characteristics have made *Ostrinia* a model for the investigation of ecological divergence and incipient speciation (Coates et al., 2018; Dopman, 2011; Lassance, 2016). Specifically, the female‐produced sex pheromone blend of E11‐ and Z11‐tetradecenyl acetate isomers (E11‐ and Z11‐14:OAc) predominates across Group III, with exception of *O. furnacalis* females that produce a blend of E12‐ and Z12‐14:OAc (Tabata & Ishikawa, 2016). Although rare, introgression was detected between *O. furnacalis* and *O. scapulalis* in eastern China (Bourguet et al., 2014), and *O. furnacalis* and *O. nubilalis* in their sympatric region of Xinjiang Uygur Autonomous Region in far western China (Wang et al., 2017). These studies indicated a degree of porosity in species barriers among closely related *Ostrinia*, and were in agreement with research which predicted that inter‐ and intraspecies variation may be the product of divergent adaptive or sexual selection at a few causal loci within a relatively homogenized genetic background (Coates et al., 2018, 2019; Kozak et al., 2019).

Additionally, univoltine and multivoltine ecotypes of *Ostrinia* respectively show obligatory and facultative entry into diapause as mature 5th instar larvae, which is triggered by a combination or interaction of cues from photoperiod and accumulated temperature exposure (Showers, 1981; Wadsworth et al., 2013; Wadsworth et al., 2020). A longer post‐diapause development (*Pdd*) time among univoltine individuals (>20 days) results in a delay in adult emergence compared to multivoltine individuals (Dopman et al., 2005; Levy et al., 2015), which may lead to location‐specific or year‐to‐year variation in degree of mating asynchrony, allochronic reproduction, and gene flow (Coates et al., 2018; Dopman et al., 2010; Wadsworth et al., 2013). Genes contributing to variance in *Pdd* are sex linked (located on the Z chromosome; Dopman et al., 2005), as are genes for diapause induction in *O. nubilalis* (Ikten et al., 2011) and *O. furnacalis* (Fu et al., 2015). Determination of *Pdd* is genetically linked to the circadian clock gene period (*per*; Levy et al., 2015). Moreover, an observed cyclical “sawtoothed” allele frequency pattern in *per* and another circadian clock gene, cryptochrome 1 (*cry1*), are spatial correlated with variation in voltinism among *O. nubilalis* populations, where estimated frequencies repeat along a northsouth cline in the United States as populations transition from one, two, and three annual reproductive generations (Levy et al., 2015). Also, sequence of two *O. nubilalis* Z chromosome positioned genomic scaffolds separately encoding per and the pigment dispersing factor receptor (*pdfr*) gene vary significantly between populations differing in *Pdd (*Kozak et al., 2019). Companion quantitative genetic analyses demonstrated that epistatic interactions between *per* and *pdfr* are explanatory of voltinism differences in *O. nubilalis* (Kozak et al., 2019 *)*, but it remains unknown whether this basis for voltinism differences are ancestral across all *Ostrinia*.

Historically, univoltine *O. furnacalis* populations pervade in eastern Jilin Province, China, as evidenced by mostly a single yearly adult flight peak (Lu et al., 2015). In contrast, multivoltine (predominantly bivoltine) populations exist at the more western locations, and sympatric populations are documented in central regions including areas around Gongzhuling (Lu et al., 1995). Univoltine populations in Jilin Province require greater accumulated temperature prior to initiation of diapause (Lu et al., 2005), show a supercooling point approximately 4°C lower (Lu et al., 1997), and have a post‐diapause development duration 20 days longer compared to bivoltine populations (Lu & Zhou, 1999). A prior population genetics study detected no significant genetic differentiation among *O. furnacalis* collection sites across eastern China, with exception of mitochondrial haplotype variation between more southern multivoltine and univoltine locations in the North (inclusive of Jilin Province; Li et al., 2014). In this study, we apply a panel of 257 single nucleotide polymorphism (SNP) markers and mitochondrial haplotype data to further investigate the roles of voltinism, geography, or other factors on *O. furnacalis* ecotype and population variation in Jilin Province, China.

## 2 MATERIAL AND METHODS

### 2.1 Sampling, haplotype sequencing, and single nucleotide polymorphism genotyping

**FIGURE 1:**
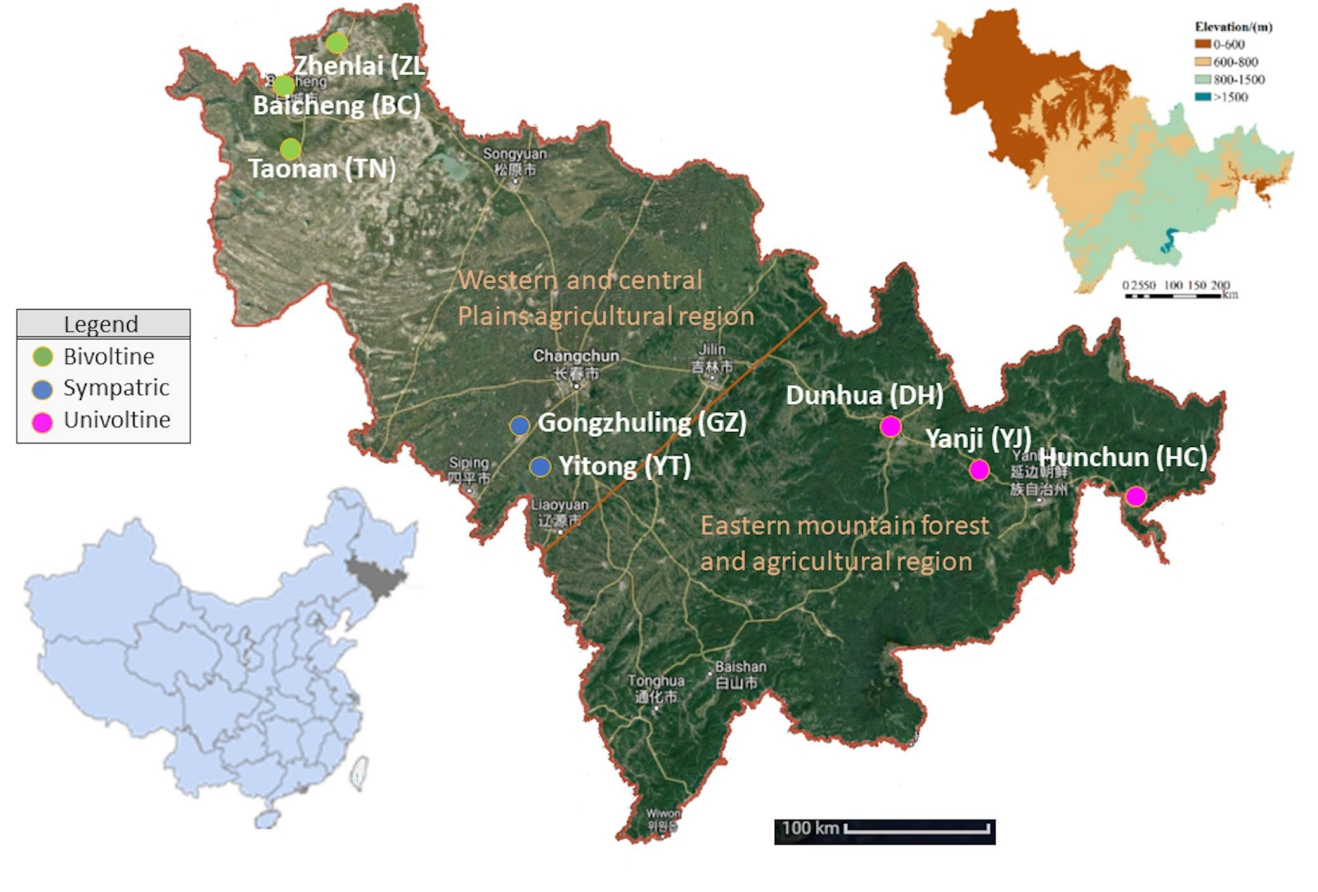
Geographic location of larval *Ostrinia furnacalis* samples from Jilin Province, P.R. China, with historical population demographics (univoltine, sympatric (mixed), or bivoltine) indicated. The species is prevalent within maize production fields across the western and central plain agricultural region, but patchy maize field habitats are common in the eastern mountain forest and agricultural region. For further location details, please refer Table 1

A total of 384 late‐instar *Ostrinia* larvae were collected from postharvest maize plants at 8 locations in Jilin Province, P.R. China, during early winter 2013 (Table 1), where voltine ecotypes were assigned based on historical prevalence (Lu et al., 1995; Lu et al., 1998). Specifically, Dunhua (DH), Hunchun (HC), and Yanji (YJ) locations have populations predominantly with a single annual mating generation (univoltine ecotype) (Table 1; Figure 1). In contrast, Taonan (TN), Baicheng (BC), and Zhenlai (ZL) locations show mostly two generations per year (bivoltine), but can show a start of a third generation under certain climatic conditions that typically does not make it through full development (two and one‐half generations per year; multivoltine ecotype). Two additional locations, Gongzhuling (GZ) and Yitong (YT), show between one and two mating generations per year and represent a mixed (sympatric) area presumably with both univoltine and multivoltine ecotypes (Table 1; Figure 1). DNA was extracted from individual larva using Ezup Column Animal Genomic DNA Purification Kits (Sangon Biotech, Shanghai, P.R. China) according to manufacturer instructions. Sample DNA quantities were estimated on a NanoDrop 2000 spectrophotometer (Thermo Scientific, Wilmington, DE, USA) and concentrations adjusted to ~10 ng/μl with nuclease‐free water. A fragment of the mitochondrial cytochrome *c* oxidase subunit I gene (COI) was PCR amplified from a subsample of 282 individuals and direct Sanger sequenced in both directions using forward and reverse primers on an ABI3700 (Applied BioSystems Inc., Foster City, CA, USA) at Sangon Biotech as described previously (Li et al., 2014; Wang et al., 2017). Individual contigs were assembled, manually trimmed of primer sequence, and unique haplotypes clustered using DnaSP v 5.10.01 (Rozas et al., 2017; 100% similarity cutoff). Resulting haplotypes were used as queries to the Barcode of Life Database (BOLD; Ratnasingham & Hebert, 2007), and accessions with the top percent identities were used for species determination. All assembled COI contig sequences were deposited in GenBank.

**TABLE 1:** *Ostrinia furnacalis* sampling in Jilin Province, P.R. China, during 2013 at locations differing in voltinism. Locations are shown on map in Figure 1

	Ecotype(s)	Geographic information				Sample counts	
		Location	Abr	Generation no.	Coordinates	COI	SNPs
Univoltine	Predominantly univoltine Eastern Jilin Province	Dunhua	DH	1	43°23′08.5″*N*	28	48
					128°15′52.4″E		
		Yanji	YJ	1	42°54′20.2″*N*	23	48
					129°25′38.0″E		
		Hunchun	HC	1	42°50′02.2″*N*	43	47
					130°22′08.6″E		
Mixed	Sympatric area; Central Jilin Province	Gongzhuling	GZ	1, 1 ½, and 2	43°34′28.5″*N*	41	48
					122°52′05.5″E		
		Yitong	YT	1, 1 ½, and 2	43°20′52.0″*N*	31	46
					125°02′04.1″E		
Multivoltine	Predominantly bivoltine Western Jilin Province.	Baicheng	BC	2 and 2 1/2	45°39′31.8″*N*	33	48
					122°46′27.1″E		
		Taonan	TN	2 and 2 1/2	45°19′11.9″*N*	42	48
					122°42′33.1″E		
		Zhenlai	ZL	2 and 2 1/2	45°47′25.4″*N*	35	47
					123°12′39.8″E		

Abbreviations: COI, mitochondrial cytochrome *c* oxidase subunit I haplotype sequences; SNPs, single nucleotide polymorphisms.

DNA extracted from the 384 collected samples was used in *Taq*I PCR‐RFLP assays to determine *O. furnacalis* or *O. nubilalis*/*O. scapulalis* species groups based on differences in the Z chromosome‐linked odorant receptor 4 (OR4) as described previously (Wang et al., 2017). Samples were also submitted for high‐throughput SNP genotyping at 318 previously described anonymous biallelic marker loci: 278 anonymous SNP markers (Coates et al., 2011) positioned on 32 *O*. *nubilalis* linkage groups (Kozak et al., 2017) and 40 Z chromosome‐linked SNP markers (Levy et al., 2015). Alternate nucleotides were detected following separation of single base extension (SBE) assay products separation on a Sequenom MassARRAY® (Sequenom, San Diego, CA, USA) located at the Center for Plant Genomics (Iowa State University, Ames, IA, USA), and genotypic calls made as described previously (Coates et al., 2011, 2013).

### 2.2 Phylogenetic relationship among mitochondrial COI haplotypes

A Bayesian information criterion (BIC) model was initially used to select the TPM2 + F + I model of sequence evolution (two‐phase model with a proportion of invariable site), which was subsequently applied for a maximum likelihood (ML)‐based phylogenetic reconstruction with 1,000 iterated ultrafast bootstrap pseudoreplicates using IQtree 1.5.6 (Minh et al., 2013; Nguyen et al., 2015). A second set of phylogenies were constructed using Bayesian Inference (BI) applied within MrBayes v.3.2 (Ronquist & Huelsenbeck, 2003). These Bayesian reconstructions used the GTR‐I‐GAMMA model (general time reversible model with a proportion of invariable sites and a gamma‐shaped distribution of rates across sites) and a parameter for among site rate heterogeneity. BI employed four simultaneous Monte Carlo Markov Chains (MCMC; one cold and three heated) with 1,000,000 generations and sampled every 500 generations. The first 25% of the data points were discarded as burn‐in. Convergence of sequences was confirmed by average standard deviation of split frequencies <0.01. The consensus trees from both ML and BI were generated using FigTree v 1.4.3 (http://tree.bio.ed.ac.uk/software/Figtree/ ).

### 2.3 Spatial genetic diversity and genetic structure

Measures of genetic diversity were calculated using both COI gene sequences and SNP marker loci. For mitochondrial COI sequences, the number of haplotypes, haplotype diversity (*h*), and nucleotide diversity (*π*) were calculated using DnaSP v 5.10.01 (Rozas et al., 2017). Analysis of molecular variance (AMOVA) was used to estimate the hierarchical partitioning of haplotype genetic variation among and within univoltine, mixed (sympatric), and bivoltine regions (Table 1; Figure 1) using Arlequin 3.5.2 (Excoffier & Lischer, 2010). Pairwise estimates of haplotype differentiation among locations, *ϕ*_ST_ (Weir & Cockerham, 1984), were generated using GenAlex (Peakall & Smouse, 2006). GenAlex was further used to: 1) carry out a principal coordinate analysis (PCoA) and then plot the scatter diagram based on factor scores along the two PCoA axes accounting for a majority of the variation, and 2) determine the correlation of genetic distance, *ϕ*_ST_/(1−*ϕ*_ST_), with geographic distance between all sample location pairs to examine adherence to an isolation‐by‐distance (IBD) model (Slatkin, 1993) using Mantel tests with significance determined following 999 permutations of the data.

Analogously, allelic diversity (mean number of alleles and effective alleles per locus) and heterozygosity (observed and expected heterozygosity under HW equilibrium) were calculated for SNP data, as well as AMOVA, PCoA, and partitioning of genetic correlation of genetic distance *F*_ST_ /(1−*F*_ST_) for adherence to an IBD model were estimated using GenAlex (Peakall & Smouse, 2006). Additionally, a Bayesian clustering approach was applied in STRUCTURE 2.3.4 (Pritchard et al., 2000) to infer the likely number of population clusters. An admixture ancestry model was implemented with correlated allele frequencies and no *a priori* population membership assignments. Simulations were iterated 10 times for each value of *K* (the number of inferred genetic clusters) from 1 to 10, with a burn‐in of 100,000 and 200,000 MCMC iterations. The optimal value of *K* was inferred from *ad hoc* posterior probability models of [Pr(X|K)] (Pritchard et al., 2000) that maximized the *ΔK* statistic (Evanno et al., 2005) as implement by STRUCTURE HARVESTER (Earl, 2012). Proportions of co‐ancestries for each individual genotype were plotted using DISTRUCT v.1.1 (Rosenberg, 2004).

### 2.4 Estimates of gene flow and haplotype exchange

Indirect estimates of gene flow between locations were generated by two approaches: 1) a classical measure of gene flow, and 2) a MCMC simulation approach. First, population genetic structure‐based gene flow was calculated according to the relationship *N*em = (1−*F*_ST_)/4*F*_ST_ (Wright, 1931), where *N*e is the effective population size of each population, and m is the immigration rate; thus, *N*em refers to the effective number of migrants per generation. This classical measure of gene flow is based on equilibrium between the forces of migration and genetic drift under the assumptions of the island model, where migration occurs among populations of equal size with symmetrical migration rates.

Secondly, maximum likelihood estimates of haplotype exchange were calculated using the coalescent‐based MCMC simulation approach with the program MIGRATE 3.2.6 (Beerli and Felsenstein, 1999). This approach takes into account the genealogical relationship of the samples and asymmetry in haplotype movement (Beerli and Felsenstein, 1999, 2001), providing parameters *θ* (= *N*eμ, where μ is the haplotype mutation rate per generation) and x*N*m, where x is 1 for mitochondrial DNA. A Bayesian approach was then used to estimate bidirectional haplotype exchange (*N*m), which was calculated from five independent runs for COI sequence data using the following Markov chain setting: sampling of 10,000 genealogies and 3 long chains with sampling of 500,000 genealogies, with burn‐in of 1,000,000 per chain.

### 2.5 Loci putative under directional selection

SNP loci that passed quality filtering criteria (*n* = 257) were applied to predict the significance of any allelic frequency differences between *O. furnacalis* samples with mitochondrial COI haplotypes found only at locations primarily with univoltine (DH, HC, and YJ) or bivoltine ecotypes (BC, TN, and ZL), and not within sympatric locations (GZ and YT). For this, an *F*_ST_ outlier approach was implemented using LOSITAN (Antao et al., 2008), which detects loci laying outside the credibility boundary based on simulation of the null distribution of *F*_ST_ across all loci using the coalescent simulation method (Beaumont & Nichols, 1996). Loci with a probability ≥0.99 are taken as showing the influence of positive selection, and probability <.01 as balancing selection. The neutral mean *F*_ST_ was estimated by removing potentially selected loci, and then, a bisectional algorithm run over repeated simulations to approximate a sample‐wise *F*_ST_. Five independent runs of 50,000 simulations were conducted under the infinite allele model (IAM) with a false discovery rate (FDR) of 10%, where FDR is defined as the expected proportion of false positives among outlier loci.

## 3 RESULTS

### 3.1 Sampling, haplotype sequencing, and single nucleotide polymorphism genotyping

*Taq*I restriction enzyme digestion of the OR4 gene fragment defined 380 specimens as *O. furnacalis* (88‐ and 104‐bp PCR amplified fragment). In contrast, amplified fragments from 4 samples were not digested and remained as intact 188‐bp products (samples HC09, YT09, YT43, and ZL24), which matched the pattern predictive of *O. nubilalis* or *O. scapulalis* (Wang et al., 2017) (Genotype data available on Dryad at https://doi.org/10.5061/dryad.x95x69pdv). Re‐running of assays for HC09, YT43, YT09, and ZL24 along with appropriate controls confirmed nondigested results (results not shown). A total of 276 of the 282 subsampled COI DNA sequences remained after filtering to remove ambiguous or poor‐quality sequences and were submitted to GenBank (accession numbers MN524233 to MN524512). Clustering of these 276 sequences resulted in 65 unique haplotypes (Table S1). Subsequent use of these 65 haplotypes as queries to the BOLD Database (current July 2019) indicated that all sequences showed top BLASTn hits with ≥97.23% similarity to accessions from *O. furnacalis*, with the exception of HC09 and YT09 which showed 98.68% similarity to accessions from *O. scapulalis* and *O. nubilalis*. Since samples HC09 and YT09 showed congruence for *O. scapulalis* between COI (Hap_65) and OR4 results (Table S2), these were excluded from analyses of *O. furnacalis* population structure. Analogously, combined data of the *O. scapulalis* OR4 genotype and *O. furnacalis* COI haplotypes of YT43 (Hap_7) and ZL24 (Hap_10) were used to define these samples as putative interspecies hybrids and thus were also excluded from further analyses.

Examination of *O. furnacalis* COI haplotypes for putative voltinism specificity (based on location history and ignoring presence at mixed (sympatric) locations) identified five putative univoltine‐specific haplotypes (Hap_01, _03, _23, _24, and _28; *n* = 32 individuals; Table S2). Only one individual from the bivoltine location, BC09, shared a putative univoltine‐specific haplotype (Hap_03). Using these same criteria, seven putative bivoltine‐specific haplotypes were identified (Hap_06, _07, _10, _16, _19, _22, and _39). These putative bivoltine‐specific haplotypes were present among 66 individuals from three historically bivoltine locations (BC, TN, and ZL), and 25 were described from mixed (sympatric) locations (GZ and YT; Figure 1; *n* = 81 total individuals; Table S2).

A multiple sequence alignment for the 64 *O*. *furnacalis* COI haplotypes resulted in a 997 bp consensus with 76 polymorphic sites, which spanned positions 1,832 to 2,831 of the *O. furnacalis* mitochondrial genome sequence AF442957.1 (Coates et al., 2005; Table S3). Out of four COI mutations, those at positions 2,021, 2,044, 2,344, and 2,746 showed high frequency differences between univoltine compared to bivoltine areas. None of these substitutions led to an amino acid change.

From among the 318 SNP loci originally developed from *O. nubilalis*, a total of 257 generated SNP genotyping results that passed filtering for all 384 individual *O. furnacalis* DNA samples. No SNP genotypes were found to be specific for univoltine or bivoltine locations. This set of genotypic dataset was used for all subsequent population genetic analyses.

### 3.2 Phylogenetic relationship among mitochondrial COI haplotypes

**FIGURE 2:**
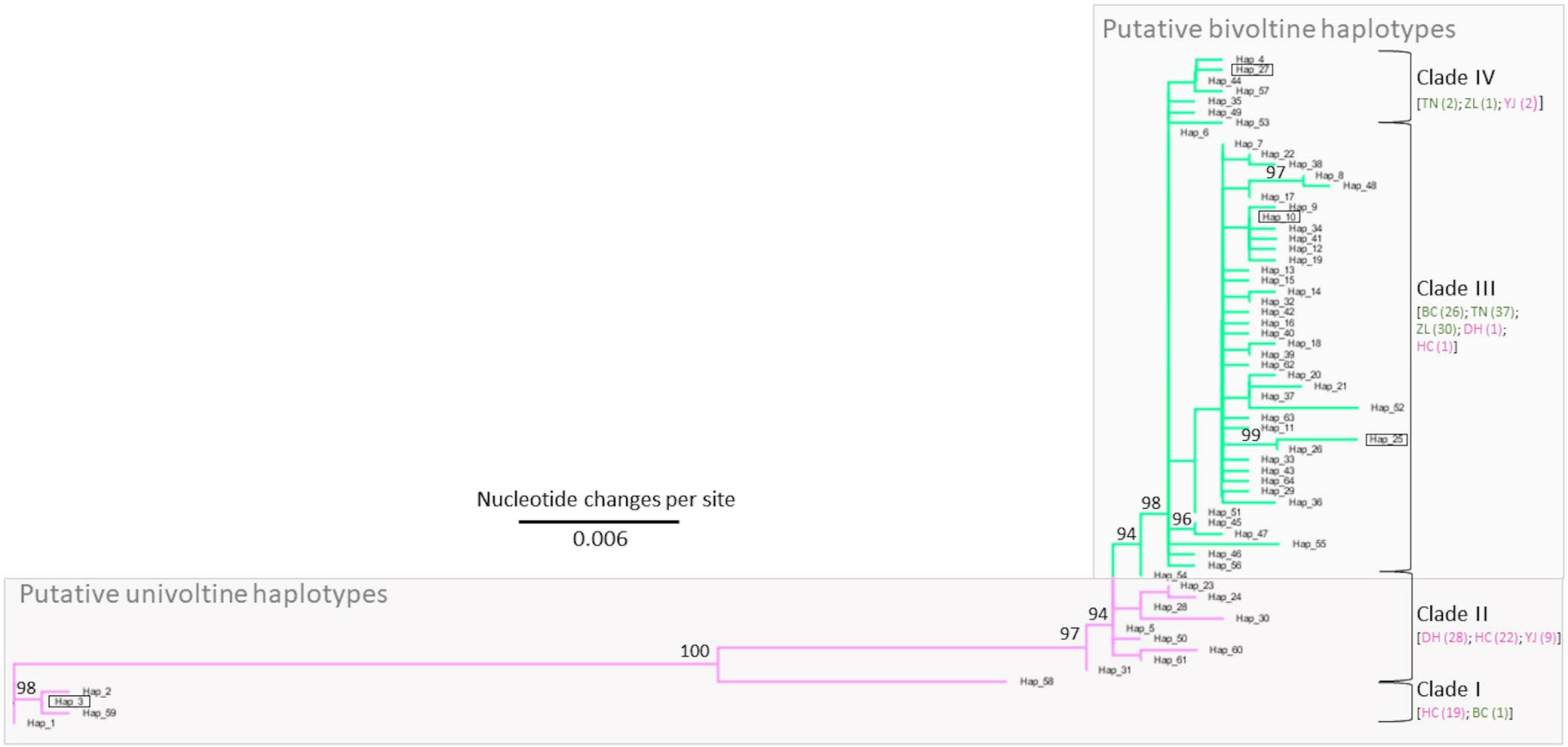
Maximum likelihood (ML)‐based phylogeny of 64 unique *Ostrinia furnacalis* mitochondrial cytochrome *c* oxidase subunit I (COI) haplotypes (Table S2) derived from 276 larval samples from 8 collection sites in Jilin Province, P.R. China, during 2013 (GenBank accession range MN524233 to MN524512). Nodes supported by >90% of 1,000 bootstrap iterations are indicated. Clades corresponding to putative bivoltine (Clades III and IV) and univoltine (Clades I and II) individuals are indicated, where counts of individuals [location (count)] are provided for each clade; haplotypes that are exceptions to this division between voltinism ecotypes between allopatric locations are enclosed in boxes

Maximum likelihood‐based phylogenetic reconstruction of 64 unique *O. furnacalis* COI haplotypes resulted in a topology with four clades (I to IV; Figure 2). Apart from samples from mixed (sympatric) locations GZ and YT, 88 of 90 individuals with 51 different haplotypes found at three bivoltine locations were clustered together within Clades III and IV. Also, 110 of 113 individuals (97.3%) within 13 haplotype groups in Clades I and II were from three univoltine locations (exception being sample BC09 with haplotype Hap_03; Table S2). The sample YT39 from the mixed (sympatric) YT location was the only instance where a haplotype (HT_02) clustered within Clades I and II was not from an individual collected at a historically univoltine location (HC, DH, or YJ; Table S2). Additional exceptions were observed in Hap_25 and Hap_27, which were comprised of only individuals from univoltine areas, but were clustered within multivoltine Clades III and IV, respectively (Figure 2). In 15 instances, only haplotypes from the mixed (sympatric) areas of GZ and YT were clustered within the bivoltine defined Clades III and IV, and were assumed to putatively comprise the bivoltine partition of those population samples. Results showed that Clades I and II were comprised of haplotypes found mostly within the historically univoltine range from eastern mountainous locations, whereas haplotypes within Clades III and IV are more shared among western historically bivoltine and mixed (sympatric) locations.

### 3.3 Spatial genetic diversity and genetic structure

**FIGURE 3:**
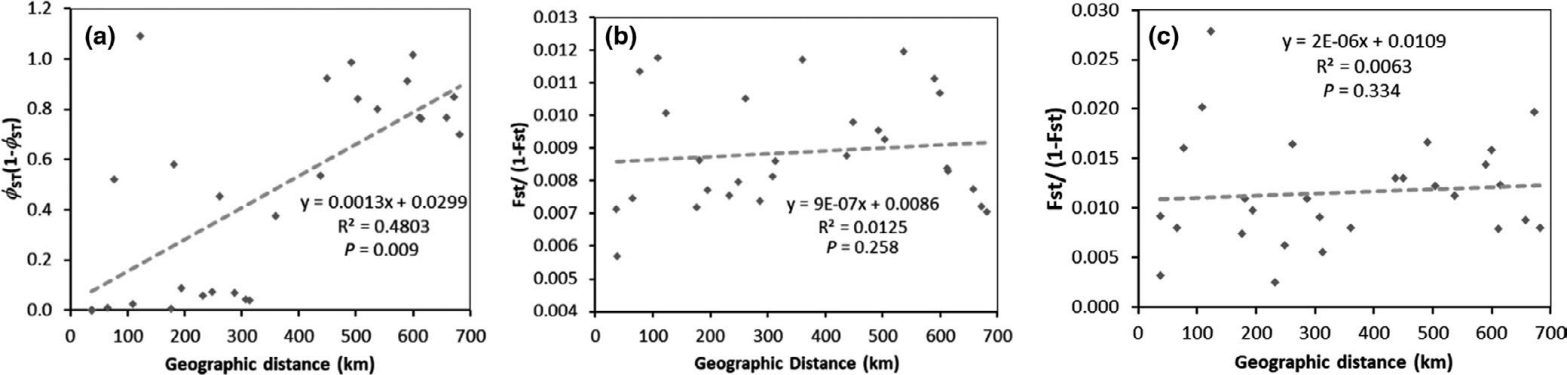
Adherence of marker data to an isolation‐by‐distance (IBD) model for pairwise variance between (a) mitochondrial cytochrome *c* oxidase subunit I (COI) haplotype and (b) 257 single nucleotide polymorphism (SNP) marker loci or C) 11 SNP marker loci putatively under selection (Table 5) collected from 8 locations in Jilin Province, China. For each, molecular variance between locations was regressed on corresponding geographic distance and significance assigned using Mantel tests (*n* = 999 permutations)

**FIGURE 4:**
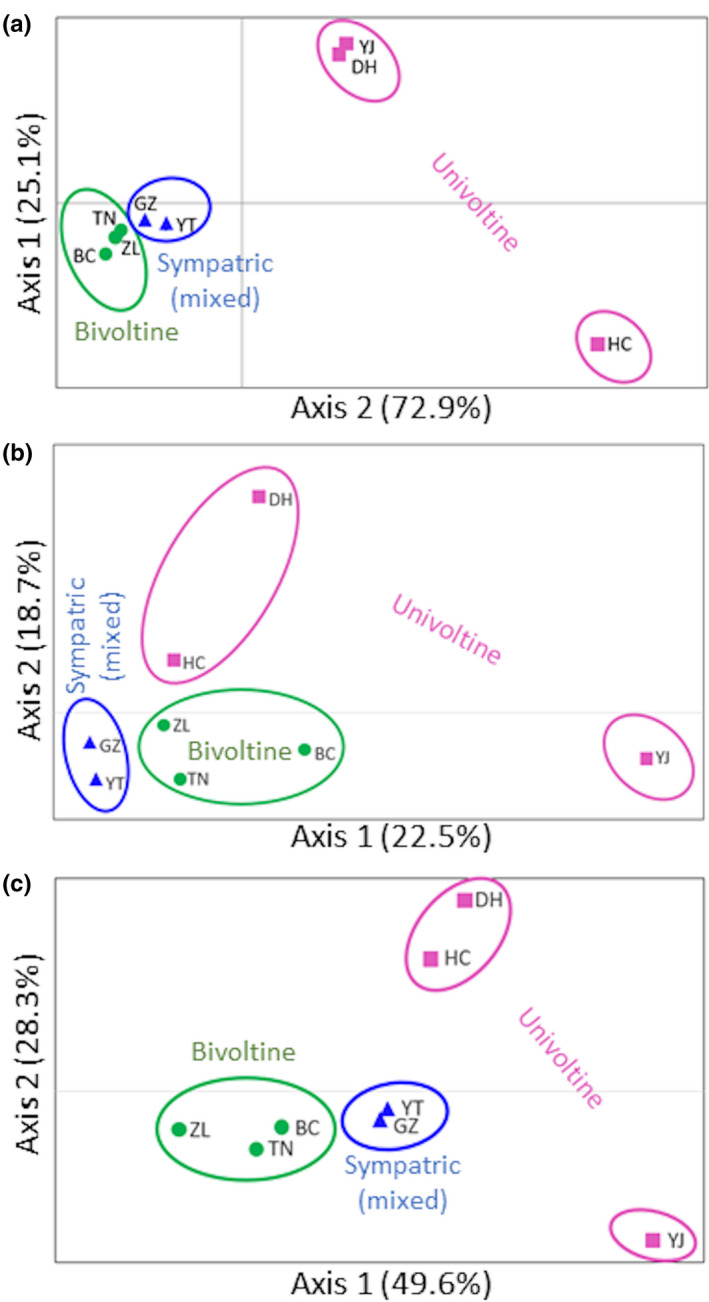
Plots of factor scores along major principal coordinates that account for the highest variation among *Ostrinia furnacalis* populations based on (a) mitochondrial cytochrome *c* oxidase subunit I (COI) haplotype, (b) variation in 257 single nucleotide polymorphism (SNP) marker loci, and (c) 11 SNP loci putatively under selection as determined by LOSITAN results

Haplotype diversity (*h*) ranged from 0.524 in DH to 0.914 in ZL and YT, and nucleotide diversity (*π*) ranged from 0.001 in YJ to 0.015 in HC for the sampled COI haplotypes across locations (Table 2). Estimates of *h* were significantly lower across locations within univoltine compared to multivoltine ecotypes (*F*_1,4_ = 19.3, *p* = .0117), or a mix of univoltine and multivoltine ecotypes (*F*_1,3_ = 11.8, *p* = .0413). Haplotype differences accounted for a significant degree of variation between univoltine, mixed, and bivoltine locations (*ϕ*_ST_ = 0.385; *p* ≤ .001), although AMOVA partitioned the greatest proportion of variance among individuals within location (61%; Table 3a). The proportion of total population variation accounted for by haplotype differences was even greater in a comparison between univoltine and bivoltine locations (*ϕ*_ST_ = 0.463; *p* ≤ .001; Table 3b), and univoltine and mixed (sympatric) locations (*ϕ*_ST_ = 0.403; *p* ≤ .001; Table S4a). In contrast, haplotype variation between bivoltine and mixed locations was low but significant (*ϕ*_ST_ = 0.060; *p* ≤ .001; Table S4b). Among mitochondrial haplotype‐based estimates of pairwise *ϕ*_ST_, 19 of 28 comparison (67.6%) surpassed the Bonferroni adjusted significance threshold (*p* ≤ .018). Among these significant comparisons, 9 (32.1%) were between univoltine and bivoltine, 6 (21.4%) were between univoltine and mix (sympatric), and two among univoltine locations (Table 4a; below diagonal). There was a significant degree of adherence to an IBD model shown by an association between haplotype variation (pairwise *ϕ*_ST_) and geographic distance (*p* = .009; Figure 3a). Principal coordinate analysis (PCoA) of COI haplotypes showed a distinct separation of locations with different predominant voltine ecotype, where the first two coordinate axes were explanatory of 98.6% of the total variation (Figure 4a). PCoA also showed that the furthest eastern location, HC, was separated from the remaining univoltine locations (DH and YJ). Factor scores also predicted that locations with mixed (sympatric) voltinism populations more closely positioned to predominantly bivoltine locations based on mitochondrial haplotypes.

**TABLE 2:** Measures of *Ostrinia furnacalis* haplotype and genotypic diversity within eight location samples from Jilin Province, P.R. China

Loc.	Voltinism	64 total COI haplotypes				257 total SNP genotypes				
		*N*	*HT* #	*h*	*π*	*N*	*N*a	*N*e	*H*o	*H*e
DH	Univoltine	28	5	0.524	0.002	43.0	1.6	1.3	0.143	0.153
YJ	Univoltine	23	7	0.696	0.001	43.8	1.6	1.3	0.142	0.149
HC	Univoltine	43	8	0.692	0.015	43.1	1.6	1.3	0.141	0.151
GZ	Mixed	41	18	0.874	0.003	44.6	1.6	1.3	0.148	0.154
YT	Mixed	31	14	0.914	0.005	42.2	1.7	1.3	0.149	0.153
BC	Bivoltine	33	12	0.896	0.004	43.2	1.7	1.3	0.145	0.150
TN	Bivoltine	42	17	0.869	0.003	45.0	1.6	1.2	0.142	0.149
ZL	Bivoltine	35	15	0.914	0.003	44.0	1.6	1.3	0.141	0.151
					Mean	43.6	1.6	1.3	0.144	0.151

Abbreviations: h, haplotype diversity; He, expected heterozygosity assuming Hardy–Weinberg equilibrium; Ho, observed heterozygosity; Na, mean number of alleles; Ne, effective number of alleles (The number of alleles in a population, weighted for their frequencies); π, nucleotide diversity.

**TABLE 3:** Analysis of molecular variance (AMOVA) of mitochondrial cytochrome *c* oxidase subunit I data between (a) three locations based on historical voltinism composition (Table 1) and (b) two locations of predominantly univoltine and bivoltine ecotypes

Source	*df*	SS	MS	Variance	% Variance	*F*‐statistic		*p*‐Value
*(a) Among regions: Univoltine, mixed (sympatric), and bivoltine locations*								
Among regions	2	193.09	96.54	0.729	0.19	*ϕ*_RT_	0.186	≤.001
Among locations	5	143.86	28.77	0.782	0.20	*ϕ*_SR_	0.245	≤.001
Among individuals	268	646.53	2.41	2.412	0.61	*ϕ*_ST_	0.385	≤.001
Total	275	983.48		3.924	1.00			
*(b) Between ecotypes: Univoltine (DH, HC, and YJ) compared to bivoltine (BC, TN, and ZL)*
Among ecotypes	1	166.66	116.65	1.276	0.26	*ϕ*_RT_	0.261	≤.001
Among locations	4	141.64	35.41	0.984	0.20	*ϕ*_SR_	0.273	≤.001
Among individuals	198	519.69	2.63	2.625	0.54	*ϕ*_ST_	0.463	≤.001
Total	203	827.99		4.885	1.00			

**TABLE 4:** Pairwise estimates of genetic diversity among eight *Ostrinia furnacalis* locations in Jilin Province, P.R. China (below diagonal), and indirect estimates of gene flow (historical number of female haplotype migrants per year, *N*m; above diagonal) based on (a) mitochondrial cytochrome *c* oxidase subunit I haplotypes (*ϕ*_ST_) and (b) 257 nuclear SNP genotypes (*F*_ST_). * indicates significant comparisons after Bonferroni correction (*p* ≤ .018). Comparisons within and between voltinism ecotypes are enclosed within dashed lined box

Abbr.	Ecotype	Univoltine			Mixed (sympatric)		Bivoltine		
		DH	YJ	HC	GZ	YT	BC	TN	ZL
*(a) ϕ_ST_ estimates from mitochondrial cytochrome c oxidase I haplotype data*									
DH	Univoltine	—	20.0	0.9	0.5	1.1	0.6	0.5	0.5
YJ	Univoltine	0.024	—	0.9	0.6	1.3	0.7	0.6	0.5
HC	Univoltine	0.367*	0.342*	—	0.7	0.9	0.7	0.5	0.7
GZ	Mixed	0.480*	0.444*	0.433*	—	78.1	8.5	5.6	6.7
YT	Mixed	0.313*	0.272*	0.348*	0.006	—	12.8	6.9	11.5
BC	Bivoltine	0.457*	0.434*	0.411*	0.056	0.038	—	Inf.	162.0
TN	Bivoltine	0.522*	0.504*	0.459*	0.081	0.067	0.000	—	55.1
ZL	Bivoltine	0.496*	0.477*	0.434*	0.070	0.042	0.003	0.009	—
*(b) F_ST_ estimates from single nucleotide polymorphism (SNP) marker data*									
DH	Univoltine	—	7.1	14.2	7.3	9.1	9.4	6.9	6.2
YJ	Univoltine	0.034*	—	14.4	9.0	5.7	245.3	11.9	7.9
HC	Univoltine	0.017*	0.034*	—	9.9	6.9	48.7	19.7	9.4
GZ	Mixed	0.033*	0.027*	0.025*	—	27.2	12.4	21.3	42.8
YT	Mixed	0.027*	0.042*	0.035*	0.009	—	7.5	8.9	12.1
BC	Bivoltine	0.026*	0.001	0.005	0.020*	0.032*	—	16.5	10.1
TN	Bivoltine	0.035*	0.021*	0.013	0.012	0.027*	0.015*	—	28.7
ZL	Bivoltine	0.033*	0.031*	0.026*	0.006	0.031*	0.024*	0.009	—

Abbreviation: Inf., infinite number of migrants based on *ϕ*_ST_ of 0.000.

In contrast, estimated levels of genetic diversity defined at 257 SNP loci were similar across all locations (Table 2). AMOVA estimated a low degree of variance among regions defined as predominantly univoltine, mixed (sympatric), and bivoltine based on genomic SNP data. Specifically, region (*F*_RT_) accounted for 0.7% of total variation (*p* ≤ .001), and relatively low and significant variation was detected among populations within region (*F*_SR_ = 0.018, *p* ≤ .001; Table S5a). Regardless, most variation was accounted for within and among individuals, together being responsible for 97.7% of total variation. A high and significant degree of local inbreeding was also predicted (*F*_IS_ = 0.481; *p* ≤ .001). AMOVA similarly showed a relatively low level of variation at SNP loci between univoltine and bivoltine locations (*F*_SR_ = 0.020 and *F*_RT_ = 0.002) along with a significant level of inbreeding (*F*_IS_ = 0.487; Table S5b). Pairwise estimates of subpopulation differentiation (*F*_ST_) among pairs of the eight locations ranged from 0.001 to 0.042, of which 21 of 28 pairwise comparisons (75%) were statistically significant after Bonferroni corrections for multiple testing (Table 4b; below diagonal). Six of nine comparisons between univoltine and bivoltine locations (66.7%) were significant, as were all comparisons between univoltine and mixed (sympatric) locations. Similarly, all intraspecific comparisons among 3 univoltine locations were low but significant. Pairwise genetic distance (*F*_ST_) estimates based on SNP marker data showed no significant association with geographic distance (*p* = .258; Figure 3b). PCoA did not differentiate locations based on pairwise *F*_ST_ estimates from among 8 geographic populations (*n* = 380 individual genotypes) based on 257 SNP loci (Figure 4b). These first 2 coordinate axes were explanatory of 41.2% of the total variation and positioned univoltine populations Dunhua (DH) and Hunchun (HC) independent of univoltine population Yanji (YJ) along coordinate axis 2 (Figure 4b).

**FIGURE 5:**
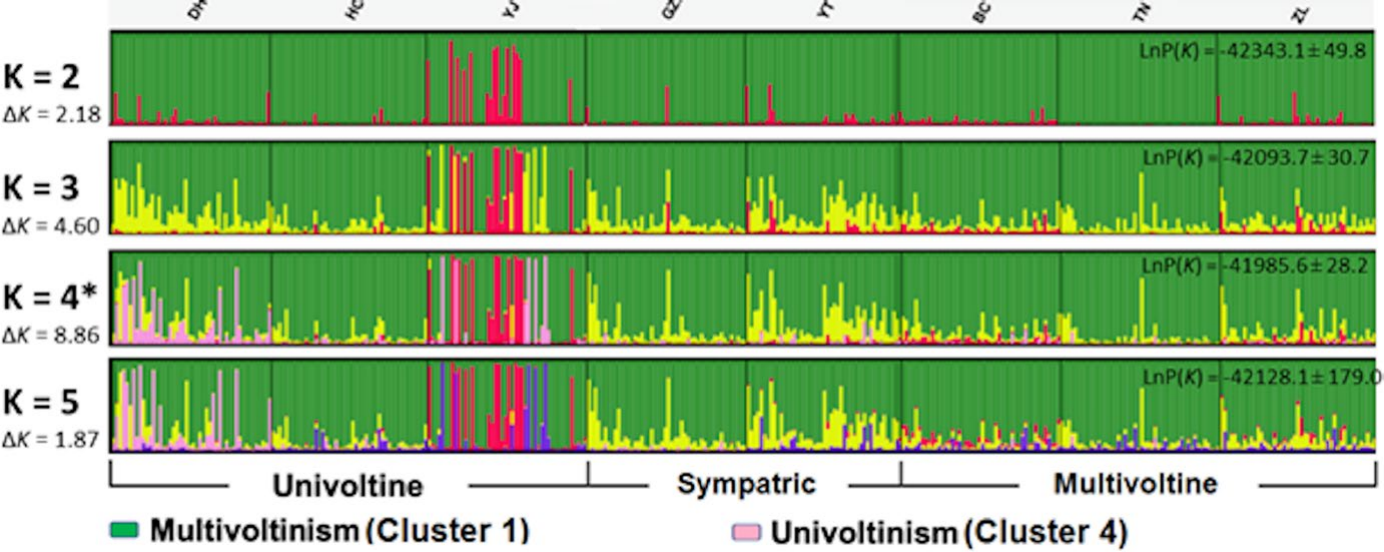
Bayesian clustering of co‐ancestries based upon 257 single nucleotide polymorphism (SNP) genotypes for 380 *Ostrinia furnacalis* larvae collected from eight locations in Jilin Province, P.R. China (Figure 1). STRUCTURE 2.3.4 estimated co‐ancestry for each individual was derived from *Q*‐matrices for iterative runs of *K* = 2 to 5 are represented proportionately within vertical lines within location. * A Δ*K* of 8.86 was maximized at *K* = 4; ln P(*K*) = −41,985.6. Abbreviations for sample locations are given in Table 1

A Bayesian clustering model implemented by STRUCTURE predicted an optimal *K* (subpopulations) = 4 (Δ*K* of 8.86 maximized with ln P(*K*) = −41985.6 ± 28.2) among the 257 SNP genotypes from 380 *O*. *furnacalis* (Figure 5). There was no overlap in the variance of ln P(*K*) from iterative estimates for each value of *K*. The proportion of estimated co‐ancestries within *Q*‐matrices at *K* = 4 showed that Cluster 4 was more prevalent among individuals within the univoltine locations (mean proportional co‐ancestry across locations 0.117 ± 0.228), but this was not significantly different compared to the mean proportion among individuals at bivoltine (0.016 ± 0.022; *F*_1,4_ = 3.658, *p* = .1284) or mixed sympatric locations (0.023 ± 0.037; *F*_1,3_ = 1.869, *p* = .2650). The univoltine location Dunhua (DH) contained the greatest mean proportion of Cluster 4 (pink) among individual co‐ancestries. Co‐ancestries among individuals from location Yanji (YJ) were distinct in that 0.258 corresponded to Cluster 2 (red), as compared to ≤0.129 among the two remaining univoltine locations and ≤0.040 among all other locations.

### 3.4 Estimates of gene flow and haplotype exchange

Estimates of the historical number of female haplotype migrants per year (*N*_m_) using the parameter *N*_m_ = [(1/*ϕ*_ST_−1)/4] (Table 3a; above diagonal) showed significantly lower estimates of *N*_m_ between univoltine and bivoltine compared to among bivoltine locations (*F*_1,10_ = 6.895, *p* = .0253). Results also indicated that haplotype exchange between univoltine and mixed locations was significantly different than between bivoltine and mixed locations (*F*_1,10_ = 43.634, *p* < .0001). Analogous estimates for *N*_m_ using variation between locations based on SNP genotype data yielded estimates of gene flow (number of migrants) that ranged from 5.7 for between YJ and YT to 245.3 between YJ and BC (Table 4b; above diagonal). Most of the population pairs generally showed a relatively moderate levels of gene flow and remained high even between univoltine and bivoltine locations (range 6.2 to 245.3).

### 3.5 Loci putatively under directional selection

Eleven SNPs were identified as candidates under directional selection, when using an *F*_ST_ outlier approach upon 257 SNP marker data from 32 individuals with 5 univoltine‐specific haplotypes and 66 individuals with 7 bivoltine‐specific haplotypes (Table S2). Among these loci, the SNP locus contig05841.408 showed a LOSITAN *p* = .958 (~ 0.989 probability of simulated *F*_ST_ < sample *F*_ST_). BLASTx search results showed significant hits for 8 of the 11 parent sequences (72.7%) to protein models of the draft *O. furnacalis* genome (PPXJ00000000.1; Table 5). For example, SNP marker contig00152.171.171 showed 98.3% identity to the *O. furnacalis* gene model XP_028171820.1 encoding a putative cytochrome P450 monooxygenase. Two of these marker loci, contig07249.251.251 and contig05841.408.408, are previously known to be positioned in *O. nubilalis* transcripts with orthologs on *B. mori* chromosome 4. Additionally, SNP marker 622_C#2 was previously designed from the Z chromosome positioned kettin gene and allele frequency variation at the autosomal marker contig07183.342.342 locus was previously correlated with latitudinal differences in *O. nubilalis* voltine ecotypes (Levy et al., 2015).

**TABLE 5:** Putative single nucleotide polymorphism (SNP) loci putatively under direction selection between *Ostrinia furnacalis* voltinism ecotypes detected using an *F*_ST_ outlier test implemented by LOSITAN. Top panel shows hits to protein models from the draft *O. furnacalis* genome (PPXJ00000000.1; unpublished)

SNP marker locus (Chr^a^)	LOSITAN			PPXJ00000000.1		
	*F*_ST_	*p*‐Value^b^	Species	Gene models	*E*‐value	Putative gene annotation
contig06857.529.529^c^ (08)	0.031	>.999	NA	NA	NA	No significant similarity found.
contig07249.251.251^c^ (04)	0.035	>.999	*Ostrinia furnacalis*	XM_028323613.1	8e^−45^	Beta−1,3‐glucan‐binding protein
contig00152.171.171^c^ (Unk)	0.112	>.999	*Ostrinia furnacalis*	XP_028171820.1	0.0	Cytochrome P450 monooxygenase
contig05937.323.323^c^ (Unk)	0.032	>.999	*Ostrinia furnacalis*	XP_028170880.1	4e^−52^	Death‐related protein 1
contig05841.408.408^c^ (04)	0.068	.958	*Ostrinia furnacalis*	XP_028175884.1	0.0	NADH dehydrogenase 1 α subunit 10
contig05821.209.209^c^ (16)	0.064	.969	*Ostrinia furnacalis*	XP_028160431.1	1e^−110^	α ‐tocopherol transfer protein isoform X1
contig05764.532.532^c^ (05)	0.133	>.999	NA	NA	NA	No significant similarity found.
contig05858.559.559^c^ (18)	0.120	.989	*Ostrinia furnacalis*	XP_028176674.1	0.0	Indole−3‐acetylaldehyde oxidase
contig07183.342.342^c^ (Aut)	0.118	>.999	NA	NA	NA	No significant similarity
mpi_B^L^ (06)	0.048	>.999	*Ostrinia nubilalis*	NA	NA	Mannose phosphate isomerase
622_C#2^L^ (Z)	0.048	>.999	*Ostrinia nubilalis*	NA	NA	Kettin

Note

Enclosed in box: significant correlation between allele frequency and latitude Levy et al. (2015).

^a^ Chr indicates chromosome position with respect to *Bombyx mori* orthologs (Levy et al., 2015).

^b^ *p*‐Value is the probability of simulated *F*_ST_ < sample *F*_ST_, where background *F*_ST_ was 0.004.

^c^ Original description by Coates et al. (2011); ^L^ Original description by Levy et al. (2015).

Corresponding correlation among differences in genetic distances (*F*_ST_) based on these 11 SNP loci as a function of geographic distances showed no adherence to an IBD model (*p* = .334; Figure 3c). Further investigation of these 11 SNP loci by PCoA of corresponding pairwise *F*_ST_ estimates between locations resulted in the clustering of predominantly bivoltine and sympatric locations into two groups along coordinate 1, where coordinate 1 accounted for 49.6% of the total variation (Figure 4c). This analysis of the 11 outlier SNP loci clustered the predominately univoltine populations Dunhua (DH) and Hunchun (HC) apart from the other predominately univoltine population, Yanji (YJ), along coordinate axis 2 that explained an additional 28.3% of the total genetic variation (Figure 4c).

## 4 DISCUSSION

The current study detected a greater degree of variation between locations defined by voltine ecotype for mitochondrial haplotypes compared to genomic SNP loci. Specifically, significant differences are observed in estimates of subpopulation divergence averaged across univoltine compared to bivoltine (*ϕ*_ST_ = 0.463; *p* < .001; Table 3a) and mixed (sympatric) locations (*ϕ*_ST_ = 0.403; *p* < .001; Table S4a) and in corresponding pairwise *ϕ*_ST_ estimates (Table 4a). Furthermore, phylogenetic analyses clustered haplotypes from predominantly univoltine locations into Clades I and II, distinct from bivoltine locations within Clades III and IV (Figure 2). PCoA analogously clustered univoltine locations apart from bivoltine and mixed (sympatric) locations (Figure 4a). Interestingly, haplotype variation was comparatively lower between bivoltine and mixed (sympatric) locations (*ϕ*_ST_ = 0.060; *p* < .001; Table S4b), which also show no significant differences in population pairwise *ϕ*_ST_ estimates (Table 4a). Prior studies ‐ show that *O. nubilalis* mitochondrial haplotype variation is low and relatively homogenized among samples collected from European locations (Hoshizaki, Washimori, Kubota, Frolov, et al., 2008), as well as between *O. nubilalis* collected on maize compared to *O. scapulalis* from dicot host plants in Poland (Piwczyński et al., 2016). When assessing voltinism or pheromone ecotypes, no haplotype variation was detected between corresponding *O. nubilalis* subpopulation in North America (Field et al., 1999). Contrasting results from a study of *O. nubilalis* detected significant haplotype variation between voltine ecotypes at locations in northern compared (univoltine) to more southern (bivoltine) regions of the Midwest United States (Coates et al., 2004). Analogously, Li et al. (2014) predicted significant haplotype variation between six *O. furnacalis* locations in northern Heilongjiang and Jilin provinces (univoltine) compared to 28 more southerly multivoltine locations in China.

Granted, voltine ecotype for collection sites was defined by geographic location in all the above studies, as similarly done in this study based on historical records (Lu et al., 1995; Lu et al., 1998), which is less precise compared to determining individual phenotype within populations. Regardless, analogous bulk population assignment to voltinism phenotype has been applied in prior studies of *Ostrinia* (Kozak et al., 2019), where these authors demonstrate coincidence of genome regions divergent between populations with different voltine ecotypes with QTL linked to *Pdd* in independent pedigree analyses. Regardless, our current results along with prior studies (Coates et al., 2004; Kozak et al., 2017) detect variation between allopatric locations differing in assigned voltine ecotype, but disentangling confounding influence of geographic (or other ecological factors) on this variance remains difficult.

There is a potentially strong effect of geographic distance on haplotype variation based on our IBD analysis (*p* = .009; Figure 3a), and both pairwise *ϕ*_ST_ estimates (Table 4a; below diagonal) and PCoA (Figure 4a). In these analyses, Hunchun (HC) is an outlier that correspondingly shows high haplotype variation when compared to all other locations (Table 4a; below diagonal) and is geographically and topologically isolated within the most eastern mountainous region. Also, a lower degree of female haplotype movement was predicted among univoltine locations in relation to other comparisons (Table 4a; above diagonal). The topography of Jilin Province in northeastern China changes from low laying plains in the west to a region of valleys separated by mountainous terrain at higher elevation and lower mean seasonal temperatures in the east. Thus, the lower movement in the east may be indicative of a patchy mitochondrial haplotype distribution within the more mountainous region where maize production fields are less contiguous and separated by highland barriers to moth movement. Thus, the dynamics of habitat patch size in combination with geographic distance and suitability of intervening habits may impact the degrees of genetic isolation (Prugh et al., 2008). This could lead to greater local fluctuations in haplotype frequencies in eastern Jilin Province based on drift, or extinction and recolonization (founder) effects. Albeit based on microsatellite makers, topographic isolation was proposed to explain the low but significant variation detected between fringe *O. nubilalis* locations within mountainous areas compared to locations within the plain regions of North America (Kim et al., 2011). This might indicate that *O. furnacalis* and *O. nubilalis* genetic structure may operate under the combined influence island (Latter, 1973) and IBD models (Slatkin, 1993) within regions of disjoined cultivated maize habitat, but this requires further investigations. Furthermore, this strong IBD among population haplotypes interjects geographic divergence as a factor that confounds the divergence presumed to be based on voltine ecotype.

In contrast to results for the above haplotype data, genomic variation is homogenized across population partitions (Table S5) and at a spatial scale when averaged across all 257 SNP (Figure 3b). When considering only genotypes from individuals with haplotypes unique to either univoltine or bivoltine individuals from allopatric locations, the LOSTAN *F*_ST_ outlier approach identified 11 of 257 SNP loci (4.3%) as putatively being under selection (Table 5). The genetic distance between locations based on this restricted set of 11 loci showed no correlation with geographic distance (Figure 3c), suggesting variation at these loci may be contingent upon voltinism or other factors. Cumulatively these results from anonymous SNP marker data are congruent with prior studies within the genus indicating that loci putatively under selection remain divergent within a genomic background that is homogenized due to high rates gene flow (Coates et al., 2019; Kim et al., 2009, 2011). Regardless, other population genetic factors, including fluctuations due to random genetic drift or linkage to other unspecified loci under selection for locally adaptive traits (Mopper & Strauss, 2013), could also be factors influencing interecotype variance at these 11 divergent loci. Despite this ambiguity, a subset of the loci was demarcated as putatively under selection, among which some correspond to markers associated with divergence between voltine ecotypes in a previous study (Levy et al., 2015).

Two of the 11 divergent *O. furnacalis* SNP loci, 622_C#2 and contig07183.342.342, were previously identified as being linked to factors contributing to *Pdd* in *O. nubilalis*. Specifically, the SNP marker, 622_C#2, detects allelic variance in the kettin, *ket*, gene located on the Z chromosome (Levy et al., 2015) and is located on the same chromosome as *Pdd* (Wadsworth & Dopman, 2015), but outside of the putative inversion that captures *Pdd* (Kozak et al., 2017). Our results showing putative differentiation at locus 622_C#2 thus may not be surprising given location on the same chromosome with *per* and *pdfr* genes that determine *Pdd* (Kozak et al., 2019). Additionally, the allele frequency variation at the autosomal marker contig07183.342.342 locus is correlated with latitudinal differences in *O. nubilalis* multivoltine ecotype across *O. nubilalis* locations transitioning from one, two, and three generations per year in the Midwest United States (Levy et al., 2015). Also, the SNP markers contig07249.251.251 and contig05841.408.408 were designed from *O. nubilalis* transcripts with predicted orthologs on chromosome 4 of *Bombyx mori* (Kozak et al., 2017). Since these two loci are in physical proximity to one another, there is a potential for effects of linkage disequilibrium and sensitivity to covariation with a genomic region putatively containing factors under the influence divergent selection between univoltine and multivoltine ecotypes. Any involvement of the 11 outlier SNP loci and their putative functional gene annotations (Table 4) are purely speculative, and deviations could be based upon population genetic factors such as drift or adaptation to other local selective pressures. Furthermore, our prediction that a subset of loci under selection between *O. furnacalis* voltine ecotypes are nonsex linked is in partial agreement with studies showing autosomal loci, such as the circadian clock gene, cryptochrome 1 (*cry1*), and the anonymous SNP locus contig05909.680, are associated with differences in generation number among multivoltine *O. nubilalis* (Levy et al., 2015). Therefore, additional genomic loci outside of *Pdd* may be involved in differences in generation number within and between voltine ecotypes, and analogous scenarios exist where modifier loci influence slight shifts in female pheromone ratios among interstrain *O. nubilalis* backcrosses (Zhu et al., 1996) that is primarily determined by the major QTL at the *pgfar* locus (Lassance et al., 2010).

Complex mechanisms and interactions, at both the ecological and genomic scale, appear to shape the contemporary structure among subpopulations within species of *Ostrinia* which differ in voltinism (Coates et al., 2018; Dopman, 2011; Kozak et al., 2017). Specifically, a few key adaptive loci are under strong divergent selection between ecotypes that differ in voltinism traits (*Pdd*; Dopman et al., 2005), where epistatic interactions between *per* and *pdfr* are major contributors in *O. nubilalis* (Kozak et al., 2019). This and prior work (Levy et al., 2015) also suggest that voltinism traits may be influenced by loci physically unlinked to *Pdd*. Furthermore, combinations or interactions of ecological, phenological, and major and minor contributing genetic factors may influence the degree of divergence within *Ostrinia* species at the population scale (Coates et al., 2019; Kozak et al., 2017; Levy et al., 2015). The prediction of significant haplotype variation between univoltine and bivoltine *O. furnacalis* in this study could be attributed to a byproduct of ecologically influenced phenology differences, degree of adult mating period asynchrony and resulting allochronic reproduction, and subsequent disparity in female mitochondrial haplotype exchange. Alternatively, random genetic drift between geographically distant or topologically divided locations could have a role. The current study provides clues into the genomic basis of voltinism variation response to different ecological scenarios, as well as the contribution of gene flow barriers to the maintenance of these divergent ecotypes. Novel insights into the possible contribution of loci outside of the major QTL for *Pdd* (Kozak et al., 2019) and voltine ecotype variation linked to *cry*1 at the population level (Levy et al. 2015) were provided via previously undescribed *F*_ST_ outlier loci. Furthermore, low female (mitochondrial haplotype)‐based estimates of genetic exchange between populations may be a consequence of mating period asynchrony or topological barriers present across the geographic ranges within this genus. Regardless of these insights, the factors and interactions culminating in the formation and maintenance of biological variants (voltine ecotypes) within natural populations remain to be completely resolved and will undoubtedly be the focus of future research endeavors.

## CONFLICT OF INTEREST

None declared.

## AUTHOR CONTRIBUTIONS

**Yangzhou Wang:** Conceptualization (equal); Formal analysis (equal); Methodology (equal); Project administration (supporting); Writing‐original draft (supporting); Writing‐review & editing (supporting). **Kyung Seok Kim:** Formal analysis (equal); Writing‐review & editing (supporting). **Qiyun Li:** Funding acquisition (equal); Project administration (equal). **Yunyue Zhang:** Investigation (supporting); Resources (supporting). **Zhen‐Ying Wang:** Funding acquisition (lead); Project administration (lead); Resources (equal); Writing‐review & editing (supporting). **Brad Steven Coates:** Conceptualization (equal); Formal analysis (equal); Methodology (equal); Project administration (supporting); Supervision (lead); Writing‐original draft (lead); Writing‐review & editing (lead).

## Supporting information

Table S1

Table S2

Table S3

Table S4

Table S5

Table S6

## Data Availability

High‐throughput genotyping data for single nucleotide polymorphism (SNP) makers genotyping data are stored in a public repository at Dryad https://doi.org/10.5061/dryad.x95x69pdv.
