# Supplementary material for: Influence of voltine ecotype and geographic distance on genetic and haplotype variation in the Asian corn borer
Source: Ecol Evol. 2021 Jul 9;11(15):10244–57. doi: 10.1002/ece3.7829 (PMC8328404; doi:10.1002/ece3.7829)
Supplement: Supplementary file 1 — Table S1 [file ECE3-11-10244-s006.pdf]

Table S1 GenBank accessions for mitochondrial cytochrome *c* oxidase subunit I gene sequences among individuals by haplotype within this study. Abbreviations for sample locations are given in Table 1.

Haplotype	Individual. ID	GenBank Accession
Hap_01	HC15	MN524233
Hap_01	HC39	MN524236
Hap_01	HC01	MN524237
Hap_01	HC04	MN524445
Hap_01	HC23	MN524447
Hap_01	HC41	MN524448
Hap_01	HC07	MN524449
Hap_01	HC26	MN524451
Hap_01	HC40	MN524452
Hap_01	HC36	MN524454
Hap_01	HC33	MN524455
Hap_01	HC19	MN524456
Hap_01	HC06	MN524512
Hap_02	YT32	MN524234
Hap_03	HC02	MN524235
Hap_03	HC35	MN524446
Hap_03	HC37	MN524450
Hap_03	HC05	MN524457
Hap_03	HC18	MN524458
Hap_04	GZ24	MN524238
Hap_05	YJ12	MN524239
Hap_05	ZL40	MN524240
Hap_05	HC46	MN524241
Hap_05	HC17	MN524242
Hap_05	TN06	MN524279
Hap_05	YJ26	MN524308
Hap_05	YJ01	MN524311
Hap_05	YJ33	MN524312
Hap_05	DH31	MN524315
Hap_05	YJ29	MN524317
Hap_05	YJ15	MN524318
Hap_05	YT06	MN524330
Hap_05	TN41	MN524379
Hap_05	GZ27	MN524381
Hap_05	HC48	MN524383
Hap_05	HC27	MN524384
Hap_05	HC11	MN524385
Hap_05	HC03	MN524386
Hap_05	HC24	MN524390
Hap_05	HC30	MN524391
Hap_05	HC10	MN524396
Hap_05	HC22	MN524399

Haplotype	Individual. ID	GenBank Accession
Hap_05	TN36	MN524400
Hap_05	HC16	MN524401
Hap_05	TN16	MN524402
Hap_05	ZL12	MN524403
Hap_05	GZ10	MN524405
Hap_05	ZL32	MN524406
Hap_05	ZL33	MN524407
Hap_05	YT17	MN524408
Hap_05	GZ36	MN524409
Hap_05	YT35	MN524410
Hap_05	HC32	MN524411
Hap_05	YT23	MN524412
Hap_05	HC34	MN524413
Hap_05	HC20	MN524414
Hap_05	HC43	MN524415
Hap_05	HC13	MN524418
Hap_05	YT22	MN524419
Hap_05	HC12	MN524425
Hap_05	HC31	MN524426
Hap_05	YJ20	MN524427
Hap_05	YT04	MN524435
Hap_05	DH02	MN524439
Hap_05	YJ17	MN524440
Hap_05	DH17	MN524441
Hap_05	TN42	MN524461
Hap_05	BC04	MN524462
Hap_05	HC42	MN524463
Hap_05	DH04	MN524464
Hap_05	YJ45	MN524465
Hap_05	DH34	MN524466
Hap_05	DH20	MN524467
Hap_05	BC03	MN524468
Hap_05	YJ39	MN524469
Hap_05	YJ27	MN524470
Hap_05	DH29	MN524471
Hap_05	DH16	MN524472
Hap_05	DH03	MN524473
Hap_05	DH05	MN524474
Hap_05	DH08	MN524475
Hap_05	DH19	MN524476
Hap_05	BC08	MN524477
Hap_05	YJ31	MN524479
Hap_05	BC07	MN524480
Hap_05	DH36	MN524481
Hap_05	DH18	MN524482

Haplotype	Individual. ID	GenBank Accession
Hap_05	DH33	MN524483
Hap_05	DH30	MN524484
Hap_05	DH35	MN524485
Hap_05	HC25	MN524486
Hap_05	DH01	MN524487
Hap_05	DH13	MN524488
Hap_06	BC45	MN524243
Hap_06	YT43	MN524271
Hap_06	YT31	MN524280
Hap_06	YT36	MN524281
Hap_06	ZL19	MN524363
Hap_06	YT12	MN524365
Hap_06	GZ32	MN524376
Hap_06	ZL22	MN524378
Hap_06	GZ15	MN524393
Hap_06	BC33	MN524395
Hap_06	ZL43	MN524398
Hap_06	YT28	MN524421
Hap_06	BC28	MN524422
Hap_06	BC43	MN524437
Hap_06	ZL48	MN524491
Hap_06	BC20	MN524492
Hap_07	TN31	MN524244
Hap_07	TN29	MN524246
Hap_07	TN17	MN524247
Hap_07	YT07	MN524250
Hap_07	ZL10	MN524251
Hap_07	ZL15	MN524253
Hap_07	BC38	MN524256
Hap_07	BC17	MN524257
Hap_07	TN44	MN524258
Hap_07	BC36	MN524262
Hap_07	YT46	MN524266
Hap_07	YT26	MN524270
Hap_07	TN01	MN524272
Hap_07	GZ22	MN524274
Hap_07	TN33	MN524284
Hap_07	ZL04	MN524286
Hap_07	TN34	MN524287
Hap_07	TN02	MN524288
Hap_07	YT34	MN524289
Hap_07	TN05	MN524292
Hap_07	GZ18	MN524320
Hap_07	ZL47	MN524324
Hap_07	ZL39	MN524337

Haplotype	Individual. ID	GenBank Accession
Hap_07	ZL37	MN524338
Hap_07	TN21	MN524344
Hap_07	ZL35	MN524348
Hap_07	TN07	MN524352
Hap_07	GZ41	MN524353
Hap_07	GZ11	MN524354
Hap_07	ZL07	MN524356
Hap_07	TN37	MN524360
Hap_07	TN19	MN524496
Hap_07	BC16	MN524502
Hap_07	TN04	MN524505
Hap_07	BC26	MN524509
Hap_07	BC19	MN524510
Hap_08	TN39	MN524245
Hap_09	TN40	MN524248
Hap_09	TN27	MN524273
Hap_10	ZL38	MN524249
Hap_10	TN18	MN524254
Hap_10	BC41	MN524260
Hap_10	BC48	MN524265
Hap_10	YT38	MN524268
Hap_10	YT13	MN524269
Hap_10	GZ14	MN524276
Hap_10	GZ23	MN524277
Hap_10	ZL42	MN524278
Hap_10	YT25	MN524282
Hap_10	HC45	MN524325
Hap_10	GZ07	MN524327
Hap_10	BC42	MN524328
Hap_10	GZ04	MN524339
Hap_10	GZ34	MN524340
Hap_10	ZL24	MN524342
Hap_10	TN23	MN524343
Hap_10	TN30	MN524347
Hap_10	GZ01	MN524349
Hap_10	ZL11	MN524350
Hap_10	BC18	MN524497
Hap_10	BC35	MN524498
Hap_10	BC13	MN524506
Hap_10	BC29	MN524507
Hap_10	TN48	MN524508
Hap_11	GZ20	MN524252
Hap_12	BC37	MN524255
Hap_12	BC46	MN524264
Hap_13	GZ09	MN524259

Haplotype	Individual. ID	GenBank Accession
Hap_14	BC40	MN524261
Hap_15	BC25	MN524263
Hap_16	TN43	MN524267
Hap_16	TN20	MN524361
Hap_16	ZL45	MN524362
Hap_17	TN08	MN524275
Hap_18	YT37	MN524283
Hap_19	TN12	MN524285
Hap_19	TN25	MN524321
Hap_19	TN32	MN524345
Hap_20	ZL17	MN524290
Hap_21	ZL05	MN524291
Hap_22	ZL18	MN524293
Hap_22	YT20	MN524322
Hap_22	ZL09	MN524329
Hap_22	ZL16	MN524346
Hap_22	BC06	MN524499
Hap_22	BC14	MN524501
Hap_22	ZL06	MN524511
Hap_23	DH24	MN524294
Hap_23	DH38	MN524295
Hap_23	DH09	MN524296
Hap_23	DH39	MN524297
Hap_23	YJ21	MN524302
Hap_24	DH27	MN524298
Hap_24	DH07	MN524299
Hap_24	DH26	MN524300
Hap_25	DH22	MN524301
Hap_26	ZL25	MN524303
Hap_27	YJ37	MN524304
Hap_28	YJ28	MN524305
Hap_28	YJ41	MN524306
Hap_28	YJ06	MN524309
Hap_28	YJ13	MN524313
Hap_28	DH12	MN524316
Hap_28	YJ22	MN524424
Hap_29	GZ08	MN524307
Hap_30	YJ40	MN524310
Hap_31	YJ43	MN524314
Hap_31	YJ42	MN524478
Hap_32	YT29	MN524319
Hap_33	TN24	MN524323
Hap_34	TN22	MN524326
Hap_34	TN15	MN524341
Hap_35	GZ02	MN524331

Haplotype	Individual. ID	GenBank Accession
Hap_36	BC39	MN524332
Hap_37	ZL23	MN524333
Hap_37	ZL03	MN524335
Hap_38	GZ25	MN524334
Hap_39	ZL31	MN524336
Hap_39	GZ31	MN524358
Hap_39	BC22	MN524503
Hap_39	BC15	MN524504
Hap_40	GZ42	MN524351
Hap_41	GZ46	MN524355
Hap_42	TN10	MN524357
Hap_43	GZ38	MN524359
Hap_44	YT01	MN524364
Hap_44	GZ35	MN524366
Hap_44	GZ39	MN524367
Hap_44	TN14	MN524368
Hap_44	GZ43	MN524371
Hap_44	YT05	MN524372
Hap_44	GZ03	MN524373
Hap_44	GZ12	MN524374
Hap_44	GZ40	MN524375
Hap_44	GZ30	MN524388
Hap_44	GZ19	MN524394
Hap_44	GZ16	MN524416
Hap_44	GZ47	MN524417
Hap_44	GZ48	MN524420
Hap_44	YT48	MN524428
Hap_44	YT24	MN524429
Hap_44	YT11	MN524431
Hap_44	GZ06	MN524432
Hap_44	YJ11	MN524489
Hap_44	GZ05	MN524490
Hap_45	TN11	MN524369
Hap_45	YT10	MN524430
Hap_46	GZ17	MN524370
Hap_47	TN26	MN524377
Hap_48	ZL13	MN524380
Hap_48	ZL46	MN524392
Hap_49	ZL34	MN524382
Hap_50	HC47	MN524387
Hap_51	YT08	MN524389
Hap_52	ZL01	MN524397
Hap_52	YT39	MN524436
Hap_52	GZ45	MN524493
Hap_53	TN13	MN524404

Haplotype	Individual. ID	GenBank Accession
Hap_54	TN46	MN524423
Hap_55	TN35	MN524433
Hap_56	YT02	MN524434
Hap_57	GZ28	MN524438
Hap_58	BC09	MN524443
Hap_59	HC29	MN524453
Hap_60	HC21	MN524459
Hap_61	HC08	MN524460
Hap_62	ZL36	MN524494
Hap_63	YT14	MN524495
Hap_64	BC31	MN524500
Hap_65	YT09	MN524442
Hap_65	HC09	MN524444
