# Supplementary material for: Influence of voltine ecotype and geographic distance on genetic and haplotype variation in the Asian corn borer
Source: Ecol Evol. 2021 Jul 9;11(15):10244–57. doi: 10.1002/ece3.7829 (PMC8328404; doi:10.1002/ece3.7829)
Supplement: Supplementary file 2 — Table S2 [file ECE3-11-10244-s005.pdf]

Table S2 Cytochrome *c* oxidase subunit I (COI) haplotypes among *Ostrinia furnacalis* larval geographic samples from Jilin Province, P.R. China. Voltinism was predicted based on prior field data (Lu, 1995). Individuals in **bold** (HC09 and YT09) show an *Ostrinia* odorant receptor 4 (OR4) species genotype that is not congruent with the mitochondrial haplotype-defined species, and represent putative *O. furnacalis*/*O. scapulalis* hybrids. Two samples defined as *O. scapulalis* (ZL24 and YT43) from both mitochondrial and OR4 data form a single group, Hap_65.

Clade*	Haplotype	Voltinism	N	Univoltine Area	Bivoltine Area	Mixed (sympatric)
I	Hap_01	Univoltine	13	HC15 HC39 HC01 HC04 HC23 HC41 HC07 HC26 HC40 HC36 HC33 HC19 HC06	*na*	*na*
I	Hap_02	Univoltine	1	*na*	*na*	YT39
I	Hap_03	Univoltine	6	HC02 HC35 HC37 HC05 HC18	BC09	*na*
I	Hap_58	Univoltine	1	*na*	*na*	1
I	Hap_59	Univoltine	1	HC29	*na*	*na*
II	Hap_05	Univoltine	73	DH01 DH02 DH03 DH04 DH05 DH08 DH13 DH16 DH17 DH18 DH19 DH20 HC25 DH29 DH30 DH31 DH33 DH34 HC34 DH35 DH36 HC03 HC10 HC11 HC12 HC13 HC16 HC17 HC20 HC22 HC24 HC27 HC30 HC31 HC32 HC42 HC43 HC46 HC48 YJ01 YJ12	*na*	*na*
II	Hap_23	Univoltine	5	DH09 DH24 DH38 DH39 YJ21	*na*	*na*
II	Hap_24	Univoltine	3	DH07 DH26 DH27	*na*	*na*
II	Hap_28	Univoltine	6	DH12 YJ06 YJ28 YJ41 YJ13 YJ22	*na*	*na*
II	Hap_30	Univoltine	1	YJ40	*na*	*na*
II	Hap_50	Univoltine	1	HC47	*na*	*na*
II	Hap_60	Univoltine	1	HC21	*na*	*na*
II	Hap_61	Univoltine	1	HC08	*na*	*na*
III	Hap_06	Bivoltine	15	*na*	BC20 BC28 BC33 BC43 BC45 ZL19 ZL22 ZL43 ZL48	YT12 YT28 YT31 YT36 **YT43** GZ15 GZ32
III	Hap_07	Bivoltine	36	*na*	BC16 BC17 BC19 BC26 BC36 BC38 TN01 TN02 TN04 TN05 TN07 TN17 TN19 TN21 TN29 TN31 TN33 TN34 TN37 TN44 ZL04 ZL07 ZL10 ZL15 ZL22 ZL35 ZL37 ZL39 ZL47	YT07 YT26 YT34 YT46 GZ11 GZ18 GZ41
III	Hap_08	Bivoltine	1	*na*	TN39	*na*

III	Hap_09	Bivoltine	2	*na*	TN40 TN27	*na*
III	Hap_10	Bivoltine	24	HC45	BC13 BC18 BC29 BC35 BC41 BC42 BC48 TN18 TN23 TN30 TN48 ZL11 **ZL24** ZL38 ZL42	YT13 YT38 YT25 GZ01 GZ04 GZ07 GZ14 GZ23 GZ34
III	Hap_11	Bivoltine	1	*na*	*na*	GZ20
III	Hap_12	Bivoltine	2	*na*	BC37 BC46	*na*
III	Hap_13	Bivoltine	1	*na*	*na*	GZ09
III	Hap_14	Bivoltine	1	*na*	BC40	*na*
III	Hap_15	Bivoltine	1	*na*	BC25	*na*
III	Hap_16	Bivoltine	3	*na*	TN20 TN43 ZL45	*na*
III	Hap_17	Bivoltine	1	*na*	TN08	*na*
III	Hap_18	Bivoltine	1	*na*	*na*	YT37
III	Hap_19	Bivoltine	3	*na*	TN12 TN25 TN32	*na*
III	Hap_20	Bivoltine	1	*na*	ZL17	*na*
III	Hap_21	Bivoltine	1	*na*	ZL05	*na*
III	Hap_22	Bivoltine	7	*na*	BC06 BC14 ZL06 ZL09 ZL16 ZL18	YT20
III	Hap_25	Univoltine	1	DH22	*na*	*na*
III	Hap_26	Bivoltine	1	*na*	TN39	*na*
III	Hap_29	Bivoltine	2	*na*	TN40 TN27	*na*
III	Hap_32	Bivoltine	1	*na*	*na*	YT29
III	Hap_33	Bivoltine	1	*na*	ZL25	*na*
III	Hap_34	Bivoltine	1	*na*	*na*	GZ08
III	Hap_39	Bivoltine	4	*na*	BC15 BC22 ZL31	GZ31
III	Hap_40	Bivoltine	1	*na*	*na*	GZ42
III	Hap_41	Bivoltine	1	*na*	*na*	GZ46
III	Hap_42	Bivoltine	1	*na*	TN10	*na*
III	Hap_43	Bivoltine	1	*na*	*na*	GZ38
III	Hap_45	Bivoltine	2	*na*	TN11	YT10
III	Hap_46	Bivoltine	1	*na*	*na*	GZ17
III	Hap_47	Bivoltine	1	*na*	TN26	*na*
III	Hap_48	Bivoltine	2	*na*	ZL13 ZL46	*na*
III	Hap_52	Bivoltine	3	*na*	ZL01	YT39 GZ45
III	Hap_55	Bivoltine	1	*na*	TN35	*na*
III	Hap_56	Bivoltine	1	*na*	*na*	YT02
III	Hap_62	Bivoltine	1	*na*	ZL36	*na*
III	Hap_63	Bivoltine	1	*na*	*na*	YT14
III	Hap_64	Bivoltine	1	*na*	BC31	*na*
III	Hap_36	Bivoltine	1	*na*	TN24	*na*
III	Hap_37	Bivoltine	2	*na*	TN15 TN22	*na*

III	Hap_38	Bivoltine	1	*na*	BC39	*na*
IV	Hap_04	Bivoltine	1	*na*	*na*	GZ24
IV	Hap_27	Univoltine	1	YJ37	*na*	*na*
IV	Hap_35	Bivoltine	1	*na*	*na*	GZ02
IV	Hap_44	Ambiguous	20	YJ11	TN14	GZ03 GZ5 GZ06 GZ12 GZ16 GZ19 GZ30 GZ35 GZ39 GZ40 GZ43 GZ47 GZ48 YT01 YT05 YT11 YT24 YT48
IV	Hap_49	Bivoltine	1	*na*	ZL34	*na*
IV	Hap_53	Bivoltine	1	*na*	TN13	*na*
IV	Hap_57	Bivoltine	1	*na*	*na*	GZ28
NA	Hap_65	*O. scapulalis*	2	HC09	*na*	YT09

* Clades defined within Maximum Likelihood (ML) analyses (Figure 2); *na* indicated when locations did not show the given haplotype within the samples.
