# Supplementary material for: Influence of voltine ecotype and geographic distance on genetic and haplotype variation in the Asian corn borer
Source: Ecol Evol. 2021 Jul 9;11(15):10244–57. doi: 10.1002/ece3.7829 (PMC8328404; doi:10.1002/ece3.7829)
Supplement: Supplementary file 4 — Table S4 [file ECE3-11-10244-s003.pdf]

Table S4 Analysis of Molecular Variance (AMOVA) of mitochondrial cytochrome *c* oxidase subunit I data between **A)** three historically univoltine locations (**Table 1**), and **B)** three historically bivoltine locations with two locations with mixed (sympatric) voltinism phenotypes.

A) Among regions: Univoltine (DH, HC & YJ) compared to mixed (sympatric; GZ & YT) locations

Source	d.f.	SS	Variance	% Variance	*F*-statistic		*P*-value
Among Regions	1	104.60	0.648	0.13	ϕ_{RT}	0.129	0.209
Among Locations	3	140.79	1.378	0.27	ϕ_{SR}	0.315	≤ 0.001
Among Individuals	161	482.87	2.999	0.60	ϕ_{ST}	0.403	≤ 0.001
Total	165	728.26	5.025	1.00			

B) Between ecotypes: Bivoltine (BC, TN & ZL) compared to mixed (sympatric; GZ & YT) locations

Source	d.f.	SS	Variance	% Variance	*F*-statistic	*P*-value	
Among Ecotypes	1	10.61	0.102	0.06	ϕ_{RT}	0.058	0.010
Among Locations	3	5.29	0.03	0.02	ϕ_{SR}	0.002	0.314
Among Individuals	177	290.49	1.641	0.92	ϕ_{ST}	0.060	≤ 0.001
Total	181	306.39	1.746	1.00			
