# Supplementary material for: Influence of voltine ecotype and geographic distance on genetic and haplotype variation in the Asian corn borer
Source: Ecol Evol. 2021 Jul 9;11(15):10244–57. doi: 10.1002/ece3.7829 (PMC8328404; doi:10.1002/ece3.7829)
Supplement: Supplementary file 5 — Table S5 [file ECE3-11-10244-s002.pdf]

Table S5 Analysis of Molecular Variance (AMOVA) of SNP genotype data between **A)** locations partitioned based on three historical voltinism compositions (univoltine, bivoltine, and mixed (sympatric); **Table 1**), and **B)** two locations of predominantly univoltine and bivoltine ecotypes.

A) Among regions: Univoltine, mixed (sympatric), and bivoltine locations

Source	d.f.	Variance	% Variance	*F*-statistic		*P*-value
Among Regions	2	0.216	0.007	F_{RT}	0.007	≤ 0.001
Among Locations	5	0.575	0.018	F_{SR}	0.018	≤ 0.001
Among Individuals	372	15.327	0.469	F_{ST}	0.024	≤ 0.001
Within Individuals	380	16.559	0.507	F_{IS}	0.481	≤ 0.001
Total	759	32.678	1.000	F_{IT}	0.493	≤ 0.001

B) Between ecotypes: Univoltine (DH, HC & YJ) compared to bivoltine (BC, TN & ZL)

Source	d.f.	Variance	% Variance	*F*-statistic		*P*-value
Among Regions	1	0.066	0.000	F_{RT}	0.002	0.016
Among Ecotypes	4	0.644	0.020	F_{SR}	0.020	≤ 0.001
Among Individuals	280	15.541	0.480	F_{ST}	0.022	≤ 0.001
Within Individuals	286	16.369	0.500	F_{IS}	0.487	≤ 0.001
Total	571	32.620	100.0	F_{IT}	0.498	≤ 0.001
