# Supplementary material for: Influence of voltine ecotype and geographic distance on genetic and haplotype variation in the Asian corn borer
Source: Ecol Evol. 2021 Jul 9;11(15):10244–57. doi: 10.1002/ece3.7829 (PMC8328404; doi:10.1002/ece3.7829)
Supplement: Supplementary file 6 — Table S6 [file ECE3-11-10244-s004.pdf]

Table S6. Pairwise Maximum Likelihood estimates of unidirectional haplotype exchange (*N*em) among *Ostrinia furnacalis* locations in Jilin Province, P.R. China (below diagonal), and indirect estimates of gene flow (*N*m; above diagonal) based on cytochrome c oxidase subunit I (COI) sequences using the coalescent-based Markov Chain Monte Carlo model (Beerli and Felsenstein 1999).

		Univoltine			Mixed (sympatric)		Bivoltine		
Abbr	Ecotype	DH	YJ	HC	GZ	YT	BC	TN	ZL
DH	Univoltine	-	2.1e+13	3.3e+13	2.1e+13	1.8e+13	2.5e+13	2.1e+13	1.8e+13
YJ	Univoltine	2.2e+17	-	5.5	3.7e+18	3.3e+18	3.6e+18	3.2e+18	4.5e+18
HC	Univoltine	85.1	1.6e+17	-	4.5	5.8	4.0	6.8	5.9
GZ	Mixed	7.2e+10	6.8e+10	1.2e+11	-	1.7e+11	11.3	5.1e+13	118.7
YT	Mixed	4.7e+13	4.5e+13	5.1e+13	4.3e+13	-	19.8	4.8e+13	105.2
BC	Bivoltine	570.1	459.5	669.2	9.8e+10	4.8e+13	-	11.3	45.2
TN	Bivoltine	5.4e+11	1.4e+12	1.4e+12	9.8e+10	3.7e+13	2.2e+12	-	4.7e+13
ZL	Bivoltine	68.8	84.6	101.6	8.2e+10	3.8e+13	107.9	73.4	-
