# Supplementary material for: Influence of voltine ecotype and geographic distance on genetic and haplotype variation in the Asian corn borer
Source: Ecol Evol. 2021 Jul 9;11(15):10244–57. doi: 10.1002/ece3.7829 (PMC8328404; doi:10.1002/ece3.7829)
Supplement: Supplementary file 3 — Table S3 [file ECE3-11-10244-s001.pdf]

Table S3 Polymorphic sites among *Ostrinia furnacalis* mitochondrial cytochrome c oxidase subunit I haplotypes identified among Jilin Province sample locations. Positions with respect to the *O. furnacalis* mitochondrial genome (AF442957.1). Substitutions are in bold and highlighted grey, with those unique to univoltine or bivoltine locations (ignoring presence in mixed areas (at positions 2,021, 2,044, 2,344 and 2,746) are highlighted yellow. Phylogenetic clades defined in Fig. 2 are indicated along with putative clustering by voltine ecotype.

		Phylogeny			Counts			AF442957.1 consensus position																																																																								
Ecotype_spec	Haplotype	Clade	Cluster	Total	univolt	bivolti	mixed	1858	1865	1879	1882	1886	1924	1939	1942	1947	1957	1987	1996	2021	2044	2057	2095	2104	2107	2113	2161	2173	2197	2212	2245	2254	2272	2276	2296	2305	2323	2326	2332	2344	2347	2350	2389	2407	2408	2431	2488	2498	2500	2401	2509	2530	2536	2551	2554	2575	2605	2614	2620	2623	2640	2641	2647	2655	2685	2707	2719	2725	2726	2731	2746	2758	2784	2788	2791	2800	2803	2811	2813	2816	2819	2824	2828
Univoltine	Hap_01	Clade I	Univoltine	13	13	0	0	T	T	A	A	C	T	T	C	G	A	A	A	T	A	C	C	G	G	T	T	A	T	A	G	T	T	T	T	A	T	T	A	T	C	A	C	C	C	C	A	G	A	T	T	T	T	A	T	A	A	C	T	C	G	A	C	T	C	T	C	G	T	T	A	C	T	C	A	A	A	C	T	T	G	A	T
	Hap_02	Clade I	Univoltine	1	0	0	1	T	T	A	A	C	T	T	C	G	A	A	A	T	A	C	C	G	A	T	T	A	T	A	G	T	T	T	T	A	T	T	A	T	C	A	C	C	C	C	A	G	A	T	T	T	T	A	T	A	A	C	T	T	G	A	C	T	C	T	C	G	T	T	A	C	T	C	A	A	A	C	T	T	G	A	T
Univoltine	Hap_03	Clade I	Univoltine	6	5	1	0	T	T	A	A	C	T	T	C	G	A	A	A	T	A	C	C	G	G	T	T	A	T	A	G	T	T	T	T	A	T	T	A	T	C	A	C	C	C	C	A	G	A	T	T	T	T	A	T	A	A	C	T	T	G	A	C	T	C	T	C	G	T	T	A	C	T	C	A	A	A	C	T	T	G	A	T
	Hap_58	Clade I	Univoltine	1	0	0	1	T	C	A	A	T	C	C	T	G	A	A	A	T	A	T	T	A	G	T	T	A	T	A	A	T	T	T	T	A	C	C	A	T	C	A	T	C	T	T	A	G	A	T	T	C	C	A	C	T	A	C	T	C	G	C	T	T	T	C	C	G	C	T	A	T	T	C	A	G	A	C	T	C	G	G	T
	Hap_59	Clade I	Univoltine	1	1	0	0	T	T	A	A	C	T	T	C	A	A	A	A	T	A	C	C	G	G	T	T	A	T	A	G	T	T	T	T	A	T	T	A	T	C	A	C	C	C	C	A	G	A	T	T	T	T	A	T	A	A	C	T	T	G	A	C	T	C	T	C	G	T	T	A	C	T	C	A	A	A	C	T	T	G	A	T
Univoltine	Hap_05	Clade II	Univoltine	73	73	0	0	T	C	A	A	T	C	C	T	G	A	A	A	T	A	C	T	A	A	C	T	A	T	G	A	T	C	T	T	A	C	T	A	T	C	A	T	C	T	T	A	G	A	T	C	T	C	A	T	T	A	T	C	C	A	T	C	T	T	C	C	A	C	C	A	C	T	T	A	A	A	C	T	T	G	A	T
Univoltine	Hap_23	Clade II	Univoltine	5	5	0	0	T	C	A	A	T	C	C	T	G	A	A	A	T	A	C	T	A	A	C	T	A	T	G	A	T	C	T	T	A	C	T	A	T	C	A	T	C	T	T	A	G	A	T	C	T	C	A	T	T	A	T	C	C	A	T	C	T	T	C	C	A	C	C	A	C	T	T	A	A	A	A	T	T	G	A	C
Univoltine	Hap_24	Clade II	Univoltine	3	3	0	0	T	C	A	A	T	C	C	T	G	A	A	A	T	A	C	T	A	A	C	T	A	T	G	A	T	C	T	T	A	C	T	A	T	C	A	T	C	T	T	A	G	A	T	C	T	C	A	T	T	A	T	C	C	A	T	C	T	T	C	C	A	C	C	A	C	A	T	A	A	A	A	T	T	G	A	C
Univoltine	Hap_28	Clade II	Univoltine	6	6	0	0	T	C	A	A	T	C	C	T	G	A	A	A	T	A	C	T	A	A	C	T	A	T	G	A	T	C	T	T	A	C	T	A	T	C	A	T	C	T	T	A	G	A	T	C	T	C	A	T	T	A	T	C	C	A	T	C	T	T	C	C	A	C	C	A	C	T	T	A	A	A	C	T	T	G	A	C
	Hap_30	Clade II	Univoltine	1	1	0	0	T	C	A	A	T	C	C	T	G	A	A	A	T	A	C	T	A	A	C	T	A	T	G	A	T	C	T	T	A	C	T	A	T	C	A	T	C	T	T	A	G	A	T	C	T	C	A	T	T	A	T	C	C	A	T	C	T	T	C	C	A	C	C	A	C	A	T	A	A	G	C	T	T	A	A	C
	Hap_50	Clade II	Univoltine	1	1	0	0	T	C	A	A	T	C	C	T	G	A	A	A	T	A	C	T	A	A	C	T	A	T	G	A	T	C	T	T	A	C	T	A	T	C	A	T	C	T	T	A	G	A	T	C	T	T	A	T	T	A	T	C	C	A	T	C	T	T	C	C	A	C	C	A	C	T	T	A	A	A	C	T	T	G	A	T
	Hap_60	Clade II	Univoltine	1	1	0	0	T	T	A	A	T	C	C	T	G	A	A	A	T	A	C	T	A	A	C	T	A	T	G	A	T	C	T	T	A	C	T	A	T	C	A	T	C	T	T	A	G	A	T	C	T	C	A	T	T	A	T	C	T	A	T	C	T	T	T	C	A	C	C	A	C	T	T	A	A	A	C	T	T	G	A	T
	Hap_61	Clade II	Univoltine	1	1	0	0	T	T	A	A	T	C	C	T	G	A	A	A	T	A	C	T	A	A	C	T	A	T	G	A	T	C	T	T	A	C	T	A	T	C	A	T	C	T	T	A	G	A	T	C	T	C	A	T	T	A	T	C	C	A	T	C	T	T	C	C	A	C	C	A	C	T	T	A	A	A	C	T	T	G	A	T
Bivoltine	Hap_06	Clade III	Bivoltine	15	0	9	6	T	C	A	A	T	C	C	T	G	A	A	A	T	G	C	T	A	A	C	T	A	T	G	A	T	C	T	T	A	C	T	A	T	C	A	T	C	T	T	A	G	A	T	C	T	C	A	T	T	A	T	C	C	A	T	C	T	T	C	C	A	C	C	G	C	T	T	A	A	A	C	T	T	G	A	T
Bivoltine	Hap_07	Clade III	Bivoltine	36	0	29	7	T	C	A	A	T	C	C	T	G	A	A	A	C	G	C	T	A	A	C	T	A	T	G	A	T	C	T	T	A	C	T	A	C	C	A	T	C	T	T	A	G	A	T	C	T	C	A	T	T	A	T	C	C	A	T	C	T	T	C	C	A	C	C	G	C	T	T	A	A	A	C	T	T	G	A	T
Bivoltine	Hap_10	Clade III	Bivoltine	24	1	14	9	T	C	A	A	T	C	C	T	G	A	A	A	C	G	C	T	A	A	C	T	A	T	G	A	T	C	T	T	A	C	T	A	C	C	A	T	C	T	T	A	G	A	T	C	T	T	A	T	T	A	T	C	C	A	T	C	T	T	C	C	A	C	C	G	C	T	T	A	A	A	C	T	T	G	A	T
Bivoltine	Hap_16	Clade III	Bivoltine	3	0	3	0	T	C	A	A	T	C	C	T	G	A	A	A	C	G	T	T	A	A	C	T	A	T	G	A	T	C	T	T	A	C	T	A	C	C	A	T	C	T	T	A	G	A	T	C	T	C	A	T	T	A	T	C	C	A	T	C	T	T	C	C	A	C	C	G	C	T	T	A	A	A	C	T	T	G	A	T
Bivoltine	Hap_19	Clade III	Bivoltine	3	0	3	0	T	C	A	A	T	C	C	T	G	A	A	A	C	G	C	T	A	A	C	T	A	T	G	A	T	C	T	T	A	C	T	A	C	C	A	T	C	T	T	A	G	A	T	C	T	T	A	T	T	A	T	C	T	A	T	C	T	T	C	C	A	C	C	G	C	T	T	A	A	A	C	T	T	G	A	T
Bivoltine	Hap_22	Clade III	Bivoltine	7	0	6	1	T	C	A	A	T	C	C	T	G	A	A	A	C	G	C	T	A	A	C	T	A	T	G	A	T	C	T	T	A	C	T	A	C	C	A	T	C	T	T	A	G	A	T	C	T	C	A	T	T	A	T	C	C	A	T	C	T	T	C	C	A	C	C	A	C	T	T	A	A	A	C	T	T	G	A	T
Bivoltine	Hap_39	Clade III	Bivoltine	4	0	3	1	T	C	A	A	T	C	C	T	G	G	A	A	C	G	C	T	A	A	C	T	A	T	G	A	T	C	T	T	A	C	T	A	C	C	A	T	C	T	T	A	G	A	T	C	T	C	A	T	T	A	T	C	C	A	T	C	T	T	C	C	A	C	C	G	C	T	T	A	A	A	C	T	T	G	A	T
	Hap_08	Clade III	Bivoltine	1	0	1	0	T	C	A	A	T	C	C	T	G	A	A	A	C	G	C	T	A	A	C	T	C	T	G	A	T	C	T	T	A	T	T	A	T	C	A	T	C	T	T	A	G	A	T	C	T	C	A	T	T	A	T	C	C	A	T	C	T	T	C	C	A	C	C	G	C	T	T	A	A	A	C	T	T	G	A	T
	Hap_09	Clade III	Bivoltine	2	0	2	0	T	C	A	A	T	C	C	T	G	A	A	A	C	A	C	T	A	A	C	T	A	T	G	A	T	C	T	T	A	C	T	A	C	C	A	T	C	T	T	A	G	A	T	C	T	T	A	T	T	A	T	C	C	A	T	C	T	T	C	C	A	C	C	G	C	T	T	A	A	A	C	T	T	G	A	T
	Hap_11	Clade III	Bivoltine	1	0	0	1	C	C	A	A	T	C	C	T	G	A	A	A	C	G	C	T	A	A	C	T	A	T	G	A	T	C	T	T	A	C	T	A	C	C	A	T	C	T	T	A	G	A	T	C	T	C	A	T	T	A	T	C	C	A	T	C	T	T	C	C	A	C	C	G	C	T	T	A	A	A	C	T	T	G	A	T
	Hap_12	Clade III	Bivoltine	2	0	2	0	T	C	A	A	T	C	C	T	G	A	A	A	C	G	C	T	A	A	C	T	A	T	G	A	T	C	T	T	A	C	T	A	C	T	A	T	C	T	T	A	G	A	T	C	T	T	A	T	T	A	T	C	C	A	T	C	T	T	C	C	A	C	C	G	C	T	T	A	A	A	C	T	T	G	A	T
	Hap_13	Clade III	Bivoltine	1	0	0	1	T	C	A	A	T	C	C	T	G	A	A	A	C	G	C	T	A	A	C	T	A	T	G	A	T	C	T	T	A	C	T	A	C	C	G	T	C	T	T	A	G	A	T	C	T	C	A	T	T	A	T	C	C	A	T	C	T	T	C	C	A	C	C	G	C	T	T	A	A	A	C	T	T	G	A	T
	Hap_14	Clade III	Bivoltine	1	0	1	0	T	C	A	A	T	C	C	T	G	A	A	A	C	G	C	T	A	A	C	T	A	T	G	A	T	C	T	T	A	C	T	A	C	C	A	T	C	T	T	G	G	A	T	C	T	T	A	T	T	A	T	C	C	A	T	C	T	T	C	C	A	C	C	G	C	T	T	A	A	A	C	T	T	G	A	T
	Hap_15	Clade III	Bivoltine	1	0	1	0	T	C	A	A	T	C	C	T	G	A	A	A	C	A	C	T	A	A	C	T	A	T	G	A	T	C	T	T	A	C	T	A	C	C	A	T	C	T	T	A	G	A	T	C	T	C	A	T	T	A	T	C	C	A	T	C	T	T	C	C	A	C	C	G	C	T	T	A	A	A	C	T	T	G	A	T
	Hap_17	Clade III	Bivoltine	1	0	1	0	T	C	A	A	T	C	C	T	G	A	A	A	C	G	C	T	A	A	C	T	A	T	G	A	T	C	T	T	A	T	T	A	C	C	A	T	C	T	T	A	G	A	T	C	T	C	A	T	T	A	T	C	C	A	T	C	T	T	C	C	A	C	C	G	C	T	T	A	A	A	C	T	T	G	A	T
	Hap_18	Clade III	Bivoltine	1	0	0	1	T	C	A	A	T	C	C	T	G	G	A	A	C	A	C	T	A	A	C	T	A	T	G	A	T	C	T	T	A	C	T	A	C	C	A	T	C	T	T	A	G	A	T	C	T	C	A	T	T	A	T	C	C	A	T	C	T	T	C	C	A	C	C	G	C	T	T	A	A	A	C	T	T	G	A	T
	Hap_20	Clade III	Bivoltine	1	0	1	0	T	T	A	A	T	C	C	T	G	A	A	A	C	G	C	T	A	A	C	T	A	T	G	A	T	C	T	T	A	C	T	A	C	C	A	T	C	T	T	A	G	A	T	C	T	C	A	T	T	A	T	C	C	A	T	C	T	T	C	C	G	C	C	G	C	T	T	A	A	A	C	T	T	G	A	T
	Hap_21	Clade III	Bivoltine	1	0	1	0	T	C	A	A	T	C	C	T	G	A	A	A	C	G	C	T	A	A	C	T	A	T	G	A	T	C	T	T	A	C	T	A	C	C	A	T	C	T	T	A	G	A	T	C	T	C	A	T	T	A	T	C	C	A	T	C	C	T	C	C	G	C	C	G	C	T	T	A	A	A	C	C	T	G	A	T
Univoltine	Hap_25	Clade III	Bivoltine	1	1	0	0	T	C	A	A	T	C	C	T	G	A	A	A	C	G	C	T	A	A	C	T	A	T	G	A	T	C	T	C	A	C	T	A	C	C	A	T	C	T	T	A	G	A	T	C	T	C	A	T	T	A	T	C	C	A	T	C	T	T	C	C	A	C	C	G	C	A	T	G	A	A	A	T	T	G	A	C
	Hap_26	Clade III	Bivoltine	1	0	1	0	T	C	A	A	T	C	C	T	G	A	A	A	C	G	C	T	A	A	C	T	A	T	G	A	T	C	T	T	A	C	T	A	C	C	A	T	C	T	T	A	G	A	T	C	T	C	A	T	T	A	T	C	C	A	T	C	T	T	C	C	A	C	C	G	C	T	T	A	A	A	A	T	T	G	A	C
	Hap_29	Clade III	Bivoltine	2	0	2	0	T	C	A	A	T	C	C	T	G	A	A	A	C	G	C	T	A	A	C	T	A	T	G	A	C	C	T	T	A	C	T	A	C	C	A	T	C	T	T	A	G	A	T	C	T	C	A	T	T	A	T	C	C	A	T	C	T	T	C	C	A	C	C	G	C	T	T	A	A	A	C	T	T	G	A	T
	Hap_32	Clade III	Bivoltine	1	0	0	1	T	C	A	A	T	C	C	T	G	A	A	A	C	G	C	T	A	A	C	T	A	T	G	A	T	C	T	T	A	C	T	A	C	C	A	T	C	T	T	G	G	A	T	C	T	C	A	T	T	A	T	C	C	A	T	C	T	T	C	C	A	C	C	G	C	T	T	A	A	A	C	T	T	G	A	T
	Hap_33	Clade III	Bivoltine	1	0	1	0	T	C	A	A	T	C	C	T	G	A	A	A	C	G	C	T	A	A	C	A	A	T	G	A	T	C	T	T	A	C	T	A	C	C	A	T	C	T	T	A	G	A	T	C	T	C	A	T	T	A	T	C	C	A	T	C	T	T	C	C	A	C	C	G	C	T	T	A	A	A	C	T	T	G	A	T
	Hap_34	Clade III	Bivoltine	1	0	0	1	T	C	A	A	T	C	C	T	G	A	A	A	C	G	C	T	A	A	C	T	A	T	G	A	T	C	T	T	G	C	T	A	C	C	A	T	C	T	T	A	G	A	T	C	T	T	A	T	T	A	T	C	C	A	T	C	T	T	C	C	A	C	C	G	C	T	T	A	A	A	C	T	T	G	A	T
	Hap_40	Clade III	Bivoltine	1	0	0	1	T	C	A	A	T	C	C	T	G	A	A	A	C	G	C	T	A	A	T	T	A	T	G	A	T	C	T	T	A	C	T	A	C	C	A	T	C	T	T	A	G	A	T	C	T	C	A	T	T	A	T	C	C	A	T	C	T	T	C	C	A	C	C	G	C	T	T	A	A	A	C	T	T	G	A	T
	Hap_41	Clade III	Bivoltine	1	0	0	1	T	C	A	A	T	C	C	T	G	A	A	A	C	G	C	T	A	A	C	T	A	T	G	A	T	C	T	T	A	C	T	A	C	C	A	T	C	T	T	A	G	A	T	C	T	T	A	T	T	A	T	C	C	A	T	C	T	T	C	C	A	T	C	G	C	T	T	A	A	A	C	T	T	G	A	T
	Hap_42	Clade III	Bivoltine	1	0	1	0	T	C	A	A	T	C	C	T	G	A	A	A	C	G	C	T	A	A	C	T	A	T	G	A	T	C	T	T	A	C	T	A	C	C	A	T	C	T	T	A	G	A	T	C	T	C	A	T	T	A	T	C	C	A	T	T	T	T	C	C	A	C	C	G	C	T	T	A	A	A	C	T	T	G	A	T
	Hap_43	Clade III	Bivoltine	1	0	0	1	T	C	A	A	T	C	C	T	G	A	A	A	C	G	C	T	A	A	C	T	A	T	G	A	T	C	T	T	A	C	T	A	C	C	A	T	C	T	T	A	A	A	T	C	T	C	A	T	T	A	T	C	C	A	T	C	T	T	C	C	A	C	C	G	C	T	T	A	A	A	C	T	T	G	A	T
	Hap_45	Clade III	Bivoltine	2	0	1	1	T	C	A	A	T	C	C	T	G	A	A	A	T	G	C	T	A	A	C	T	A	T	G	A	T	C	T	T	A	C	T	A	T	C	A	T	C	T	T	G	G	A	T	C	T	C	A	T	T	A	T	C	C	A	T	C	T	T	C	C	A	C	C	G	C	T	T	A	A	A	C	T	T	G	A	T
	Hap_46	Clade III	Bivoltine	1	0	0	1	T	C	A	A	T	C	C	T	G	A	A	A	T	G	C	T	A	A	C	T	A	T	A	A	T	C	T	T	A	C	T	A	T	C	A	T	C	T	T	A	G	A	T	C	T	C	A	T	T	A	T	C	C	A	T	C	T	T	C	C	A	C	C	G	C	T	T	A	A	A	C	T	T	G	A	T
	Hap_47	Clade III	Bivoltine	1	0	1	0	T	C	A	A	T	C	C	T	G	A	A	A	T	G	C	T	A	A	C	T	A	T	G	A	T	C	T	T	A	C	T	A	T	T	A	T	C	T	T	G	G	A	T	C	T	C	A	T	T	A	T	C	C	A	T	C	T	T	C	C	A	C	C	G	C	T	T	A	A	A	C	T	T	G	A	T
	Hap_48	Clade III	Bivoltine	2	0	2	0	T	C	A	A	T	C	C	T	G	A	A	A	T	G	C	T	A	A	C	T	C	T	G	A	T	C	T	T	A	T	T	A	T	C	A	T	C	T	T	A	G	A	T	C	T	C	A	T	T	A	T	C	C	A	T	C	T	T	C	C	A	C	C	G	C	T	T	A	A	A	C	T	T	G	A	T
	Hap_52	Clade III	Bivoltine	3	0	1	2	T	C	A	A	T	C	C	T	G	A	A	A	T	G	C	T	A	A	C	T	A	T	A	A	T	C	T	T	A	C	T	A	C	C	A	T	C	T	T	A	G	A	C	C	T	C	A	T	T	A	T	T	C	A	T	C	T	T	C	C	G	C	C	G	C	T	T	A	A	A	C	T	T	G	A	T
	Hap_55	Clade III	Bivoltine	1	0	1	0	T	C	A	A	T	C	C	T	G	A	A	A	T	G	C	T	A	A	C	T	A	T	G	A	T	C	T	T	A	C	T	A	T	C	A	T	T	T	T	A	G	A	T	C	T	C	G	T	T	G	T	C	T	A	T	C	T	T	C	C	A	C	C	G	C	T	T	A	A	A	C	T	T	G	A	T
	Hap_56	Clade III	Bivoltine	1	0	0	1	T	C	T	A	T	C	C	T	G	A	A	A	T	G	C	T	A	A	C	T	A	T	G	A	T	C	T	T	A	C	T	A	T	C	A	T	C	T	T	A	G	A	T	C	T	C	A	T	T	A	T	C	C	A	T	C	T	T	C	C	A	C	C	G	C	T	T	A	A	A	C	T	T	G	A	T
	Hap_62	Clade III	Bivoltine	1	0	1	0	T	C	A	G	T	C	C	T	G	A	A	A	C	G	C	T	A	A	C	T	A	T	G	A	T	C	T	T	A	C	T	A	C	C	A	T	C	T	T	A	G	A	T	C	T	C	A	T	T	A	T	C	C	A	T	C	T	T	C	C	A	C	C	G	C	T	T	A	A	A	C	T	T	G	A	T
	Hap_63	Clade III	Bivoltine	1	0	0	1	T	C	A	A	T	C	C	T	G	G	A	A	C	G	C	T	A	A	C	T	A	T	G	A	T	C	T	T	A	C	T	A	C	C	A	T	C	T	T	A	G	A	T	C	T	C	A	T	T	A	T	C	C	A	T	C	T	T	C	C	A	C	C	G	C	T	T	A	A	A	C	T	T	G	A	T
	Hap_64	Clade III	Bivoltine	1	0	1	0	T	C	A	A	T	C	C	T	G	A	A	A	C	G	C	T	A	A	C	T	A	T	G	A	T	C	T	T	A	C	T	C	C	C	A	T	C	T	T	A	G	A	T	C	T	C	A	T	T	A	T	C	C	A	T	C	T	T	C	C	A	C	C	G	C	T	T	A	A	A	C	T	T	G	A	T
	Hap_04	Clade IV	Bivoltine	1	0	0	1	T	C	A	A	T	C	T	T	G	A	A	A	T	G	C	T	A	A	C	T	A	T	G	A	T	C	T	T	A	C	T	A	T	C	A	T	C	T	T	A	G	A	T	C	T	C	A	T	T	A	T	C	C	A	T	C	T	T	C	C	A	C	T	G	C	T	T	A	A	A	C	T	T	G	A	T
	Hap_27	Clade IV	Bivoltine	1	1	0	0	T	C	A	A	T	C	C	T	G	A	A	A	T	G	C	T	A	A	C	T	A	T	G	A	T	C	T	T	A	C	T	A	T	C	A	T	C	T	T	A	G	A	T	C	T	C	A	T	T	A	T	C	C	A	T	C	T	T	C	C	A	C	T	G	C	T	T	A	A	A	C	T	T	G	A	C
	Hap_35	Clade IV	Bivoltine	1	0	0	1	T	C	A	A	T	C	C	T	G	A	A	A	T	G	C	T	G	A	C	T	A	T	G	A	T	C	T	T	A	C	T	A	T	C	A	T	C	T	T	A	G	A	T	C	T	C	A	T	T	A	T	C	C	A	T	C	T	T	C	C	A	C	C	G	C	T	T	A	A	A	C	T	T	G	A	T
	Hap_44	Clade IV	Bivoltine	20	1	1	18	T	C	A	A	T	C	C	T	G	A	A	A	T	G	C	T	A	A	C	T	A	T	G	A	T	C	T	T	A	C	T	A	T	C	A	T	C	T	T	A	G	A	T	C	T	C	A	T	T	A	T	C	C	A	T	C	T	T	C	C	A	C	T	G	C	T	T	A	A	A	C	T	T	G	A	T
	Hap_49	Clade IV	Bivoltine	1	0	1	0	T	C	A	A	T	C	C	T	G	A	A	A	T	G	C	T	A	A	C	T	A	T	G	A	T	C	C	T	A	C	T	A	T	C	A	T	C	T	T	A	G	A	T	C	T	C	A	T	T	A	T	C	C	A	T	C	T	T	C	C	A	C	C	G	C	T	T	A	A	A	C	T	T	G	A	T
	Hap_53	Clade IV	Bivoltine	1	0	1	0	T	C	A	A	T	C	C	T	G	A	A	A	T	G	C	T	A	A	C	T	A	T	G	A	T	C	T	T	A	C	T	A	T	C	A	T	C	T	T	A	G	G	T	C	T	C	A	T	T	A	T	C	C	A	T	C	T	T	C	T	A	C	C	G	C	T	T	A	A	A	C	T	T	G	A	T
	Hap_57	Clade IV	Bivoltine	1	0	0	1	T	C	A	A	T	C	C	T	G	A	A	A	T	G	C	T	A	A	C	T	T	T	G	A	T	C	T	T	A	C	T	A	T	C	A	T	C	T	T	A	G	A	T	C	T	C	A	T	T	A	T	C	C	A	T	C	T	T	C	C	A	C	T	G	C	T	T	A	A	A	C	T	T	G	A	T
	Hap_31	Clade II	Bivoltine		Yangzhou fill in			T	C	A	A	T	C	C	T	G	A	A	A	T	A	C	T	A	A	C	T	A	T	A	A	T	C	T	T	A	C	T	A	T	C	A	T	C	T	T	A	G	A	T	C	T	C	A	T	T	A	T	C	C	A	T	C	T	T	C	C	A	C	C	A	C	T	T	A	A	A	C	T	T	G	A	T
	Hap_36	Clade III	Bivoltine	1	0	1	0	T	C	A	A	T	C	C	T	G	A	A	G	C	G	C	T	A	A	C	T	A	T	G	A	T	C	T	T	A	C	T	A	C	C	A	T	C	T	T	A	G	A	T	C	T	C	A	T	T	A	T	C	C	A	T	C	T	T	C	C	A	C	T	G	C	T	T	A	A	A	C	T	T	G	A	T
	Hap_37	Clade III	Bivoltine	2	0	2	0	T	C	A	A	T	C	C	T	G	A	A	A	C	G	C	T	A	A	C	T	A	T	G	A	T	C	T	T	A	C	T	A	C	C	A	T	C	T	T	A	G	A	T	C	T	C	A	T	T	A	T	C	C	A	T	C	T	T	C	C	G	C	C	G	C	T	T	A	A	A	C	T	T	G	A	T
	Hap_38	Clade III	Bivoltine	1	0	1	0	T	C	A	A	T	C	C	T	G	A	A	A	C	A	C	T	A	A	C	T	A	T	G	A	T	C	T	T	A	C	T	A	C	C	A	T	C	T	T	A	G	A	T	C	T	C	A	T	T	A	T	C	C	A	T	C	T	T	C	C	A	C	C	A	C	T	T	A	A	A	C	T	T	G	A	T
	Hap_51	Clade III	Bivoltine		Yangzhou fill in			T	C	A	A	T	C	C	T	G	A	A	A	T	G	C	T	A	A	C	T	A	T	G	A	T	C	T	T	A	C	T	A	C	C	A	T	C	T	T	A	G	A	T	C	T	C	A	T	T	A	T	C	C	A	T	C	T	T	C	C	A	C	C	G	C	T	T	A	A	A	C	T	T	G	A	T
	Hap_54	Clade III	Bivoltine		Yangzhou fill in			T	C	A	A	T	C	C	T	G	A	A	A	T	G	C	T	A	A	C	T	A	T	G	A	T	C	T	T	A	C	T	A	T	C	A	T	C	T	T	A	G	A	T	C	T	C	A	T	T	A	T	C	C	A	T	C	T	T	C	C	A	C	C	A	C	T	T	A	A	A	C	T	T	G	A	T
